# Supplementary material for: Forest fire size amplifies postfire land surface warming
Source: Nature. 2024 Sep 25;633(8031):828–34. doi: 10.1038/s41586-024-07918-8 (PMC11424489; doi:10.1038/s41586-024-07918-8)
Supplement: Supplementary file 1 — Supplementary Notes 1–6, Figs. 1–28 and Tables 1–7. [file 41586_2024_7918_MOESM1_ESM.docx]

Supplementary Information for

Forest fire size amplifies postfire land surface warming

Jie Zhao, Chao Yue^✉^, Jiaming Wang, Stijn Hantson, Xianli Wang, Binbin He, Guangyao Li, Liang Wang, Hongfei Zhao, Sebastiaan Luyssaert.

Correspondence to: chaoyuejoy@gmail.com

Supplementary Text 1 | Robustness of the amplification by fire size in postfire surface warming in summer

Several regional fire patch datasets (detailed below) are available for the Canadian and Alaskan boreal forests (Supplementary Fig. 9). The regional datasets are produced by local data providers and are therefore considered to have better quality and accuracy than the fire patch datasets with global coverage (GFA or GlobFire). Different datasets were used to examine the robustness of the reported linear relationship between postfire surface radiometric temperature change (mainly dominated by a warming effect) and fire size.

Two datasets for the Canadian and Alaskan boreal forests generated by the Arctic-Boreal Vulnerability Experiment (ABoVE) project were used. The ABoVE is a NASA Terrestrial Ecology Program field campaign conducted in Alaska and western Canada starting in 2015. The two fire patch datasets were generated by combining satellite-observed burn date (or burn severity) information and fire scar information from government agencies. One was based on Landsat images (https://daac.ornl.gov/ABOVE/guides/ABoVE_Fire_Severity_dNBR.html) (hereafter referred to as ABoVE-Landsat) while the other was based on the MODIS active fire count product (https://daac.ornl.gov/ABOVE/guides/Wildfires_Date_of_Burning.html) (hereafter referred to ABoVE-MODIS). Both ABoVE-Landsat and ABoVE-MODIS used fire perimeter information from the Alaskan Interagency Coordination Center (AICC) and Natural Resources Canada. Hence, using these products is essentially using the government agency data, the same data as used for Canada in Extended Data Fig. 10.

The third product used was the Monitoring Trends in Burn Severity (MTBS) dataset, which was based on Landsat images and covers both Alaska and the continental USA. This product was also utilized to examine temporal trends in burned area and fire size for the whole USA as shown in Extended Data Fig. 10. In this supplementary analysis, however, only the fire patches in Alaska were used. The two global fire-patch datasets, both based on the MCD64A1 burned-area data, were GFA and GlobFire (for GlobFire please refer to Supplementary Text 5).

These five products cover roughly the same area and could, therefore, be used to examine the robustness of the relationship between summer postfire surface temperature change (ΔΤ) and fire size. First, in terms of fire size distribution, the five datasets could be broadly divided into two groups (Supplementary Fig. 10a). The two global datasets showed a dominance of fires with a size slightly larger than 1 km^2^ (about 10^0.2^ km^2^ or 1.6 km^2^), whereas the three regional datasets showed a dominance of fires with a size of 10–100 km^2^, being one or two orders of magnitudes larger than the value from GFA or GlobFire, suggesting that both GFA and GlobFire very likely underestimate fire size for this region. Note that GlobFire reported the largest probability density of small fires of all the datasets and therefore likely underestimates fire size even more severely than GFA.

All five datasets showed broad surface warming in summer one year after fire (the first-column panels of Supplementary Fig. 11). Despite their differences in fire size distribution, all datasets showed significant linear relationships between postfire summer ΔΤ and fire size (with all p-values being smaller than 0.05, Supplementary Figs. 10b–10f), suggesting enhanced surface warming with fire size. Regressions using the three regional datasets of ABoVE-MODIS, ABoVE-MODIS and MTBS clearly show better goodness-of-fit (R^2^=0.35–0.46) than the two global datasets (R^2^=0.08–0.11), probably due to their better data quality. The differences in fire size distribution among different datasets influenced the derived β_ΔΤ_: the two global datasets, dominated by small fires, reported a lower β_ΔΤ_ (0.40–0.51) than the regional datasets (0.61–0.69), which were dominated by larger fires. Given that all five datasets covered roughly the same region and hence their underlying fire regimes should be the same, it seems likely that the two global datasets, both dominated by small fires, may have erroneously divided a large fire into several smaller ones, and as a result the overall ΔT for small fires was inflated, explaining their lower β_ΔT_ values.

The linear relationship between postfire summer ΔΤ and fire size also holds when it is examined for each 2º grid cell (Supplementary Fig. 11). The results of spatially explicit regressions between summer ΔΤ and fire size confirmed the results of the regressions made by pooling all data together across the space: the regional datasets show better goodness-of-fit (R^2^) and higher values of β_ΔΤ_ than the global datasets (Supplementary Fig. 12).

In conclusion, the reported amplification of postfire summer ΔΤ by fire size using GFA was robust but β_ΔΤ_ was underestimated because GFA underestimated fire size for this region. This implies that in reality the impact of fire size on postfire surface warming in summer is more likely to be higher than reported in the main text.

Supplementary Text 2 | Winter surface temperature change after fire and the snow effect

ΔΤ in winter was dominated by radiative processes, resulting in surface radiometric cooling consistent with an increase in surface albedo (Supplementary Figs. 13a, 13e). The importance of radiative processes in driving winter surface cooling is also demonstrated by the fact that only the changes in surface albedo, reflected shortwave radiation and surface radiometric temperature show some dependence on fire size (mainly limited to North America), whereas changes in the other biogeophysical variables show negligible size dependence (Supplementary Fig. 13).

The winter cooling effect by fire and its change with fire size were suspected to be affected by the presence of snow. To test this hypothesis, latitudinal patterns of snow cover, postfire change in surface albedo (Δα), surface temperature change (ΔΤ) and its fire-size sensitivity (β_ΔΤ_) one year after fire were examined. Note our main study spatial domain is northern temperate and boreal forests above 40ºN but for this specific analysis, in order to include regions with less snow in winter, the spatial domain was extended to 30ºN and included North America only.

MODIS Normalized Difference Snow Index (NDSI) showed that winter snow cover reduced with decreasing latitude to an almost non-existent level at 30ºN (Supplementary Fig. 14). Accordingly, Δα increased most prominently at 70ºN and reduced with latitude to almost zero at 30ºN. Winter ΔT then transitioned from a pronounced cooling effect in the region of 55ºN–70ºN to zero cooling at 30ºN. The observed consistent decreases in Δα, ΔΤ and snow cover with decreasing latitude hence suggest a strong regulation by snow in the postfire winter biophysical changes. Finally, β_ΔΤ_ showed similar latitudinal patterns with ΔT, with higher values found in high-latitude zones and lower values in mid-latitude zones.

The crucial role of snow cover in driving the observed winter surface cooling in northern forests was further confirmed by an additional analysis of winter surface temperature change one year after fire in Australian forests (Supplementary Fig. 3g–3i, Supplementary Table 1): Australian fires lead to surface warming even in winter because of the absence of the winter albedo effect due to the almost non-existent snow cover.

Supplementary Text 3 | Surface temperature change following fire and harvest in Canadian forest

The observed enhanced postfire change in surface radiometric temperature (ΔΤ) with fire patch size is hypothesized to result from two effects: (1) the ‘direct size effect’, which is related to postfire landscape heterogeneity that directly depends on the spatial extent of a burned patch. That is, following larger fires, a spatially more homogeneous landscape will lead to smaller surface roughness, reducing near-surface turbulence and hampering surface heat dissipation through evaporative cooling, thus resulting in increased warming; and (2) the ‘fire behaviour effect’, which refers to increasing fire intensity and/or fire severity co-occurring with increasing fire size, both being driven by the more favourable fire weather which underlies a larger fire.

The presence of a direct size effect can be demonstrated by considering forest disturbance where the disturbance patch size is independent from its intensity. We reasoned that this is the case for forest harvest where both small and large final clearcuts remove the same proportion of trees, i.e., close to 100%. For such disturbances the change in surface temperature (ΔΤ) as a function of harvest patch size would represent the direct size effect.

We hypothesized that if the direct size effect exists, then ΔΤ following forest harvest should increase with harvest patch size. We further hypothesized that for a region with both fire and harvest disturbances, given that ΔΤ following fire disturbance is subject to an additional ‘fire behaviour effect’, it’s to be expected that β_ΔΤ_ following fire (β_ΔΤ, fire_) will be larger than β_ΔΤ_ following harvest (β_ΔΤ, harvest_), with their difference indicating an effect of fire behaviour. The difference is not necessarily equal to the effect of fire behaviour, as this approach does not allow quantifying possible interactions between the direct size effect and the fire-behaviour effect.

The forest disturbance dataset developed by White et al.^1^, which covers the whole of Canada for 1985–2015, provides suitable data for testing our hypotheses. The White et al. dataset provides stand-replacing forest loss events on a 30m spatial resolution due to stand-replacing wildfires (the dominant fire regime over Canada) or clearcut forest harvest (i.e., 100% of forest over a given 30m pixel was removed)^1^. The availability of large scale LST data with a 30m resolution would make the analysis straightforward. However, we are not aware of any such data which are credible, cover a long-term period, and have been widely used in analyses of LST change associated with land cover or forest cover change. Instead, we used MODIS-based LST data (with a resolution of 1km or 0.05º) which have been widely used in previous studies^2-6^.

The land surface temperature observation data for 2002–2016, based on MODIS and used for the analysis presented in the main text, has an original resolution of 1km but was resampled to 500m resolution, to be consistent with the spatial resolution of the MODIS burned area data used (see Methods of the main text). The dataset of White et al.^1^ was processed to have the same resolution as MODIS burned area data used in the main text, again to be consistent with the spatial resolution of the analysis described in the main text.

The ground fraction subject to stand-replacing forest fire or clearcut harvest within each MODIS-equivalent 500m pixel (denoted as ‘disturbance fraction’ or F_dist_) was first calculated. All 500m pixels with F_dist_ exceeding a threshold of 20% (a value chosen to be to be consistent with the threshold used in the main text to identify the control unburned pixels to derive ΔΤ_res_, see Equation (2) and the associated methods in the main text) were considered as ‘disturbance pixels’. Pixels within 500 meters of each other were considered to belong to the same fire or harvest patch. The mean value of F_dist­_ was calculated for each patch. To be consistent with the analysis in the main text, only patches > 1km^2^ were included in the analysis here.

**Approach 1: multiple linear regression analysis**

An initial examination shows that both ΔΤ in summer and F_dist_ increase significantly with the logarithm of patch size for both fire and harvest disturbances (Supplementary Fig. 15). The value of β_ΔΤ, harvest_ being significantly different from zero thus supports our first hypothesis. Although β_ΔΤ, fire_ is greater than β_ΔΤ, harvest_, for a given patch size, F_dist_ is also greater for fire than for harvest. Subsequently, a multiple linear regression model was applied to compare β_ΔΤ, fire_ and β_ΔΤ, harvest_ after controlling for F_dist_. The multiple linear regression model used ΔΤ as the dependent variable, with log_10_(size), F_dist_, and disturbance type (‘Type’, a categorical variable of ‘fire’ or ‘harvest’) as the independent variables (Supplementary Table 3).

The regression analysis shows that ΔΤ increases with F_dist_ for both fire and harvest, with the slope of F_dist_ showing no statistical difference (*p*=0.14) between fire and harvest. It is plausible that the response of ΔΤ to F_dist_ is similar for fire and harvest, because it is an effect whose strength is expected to be proportional to area (Supplementary Table 3). Next, β_ΔΤ, harvest_ was found to differ significantly from zero, indicating a direct size effect on ΔΤ for forest harvest, even after controlling for co-varying disturbance fraction (Supplementary Table 3). Finally, β_ΔΤ, fire_ was found to be significantly larger than β_ΔΤ, harvest_ (*p*<0.01, Supplementary Table 3), implying an additional fire behaviour effect, such as increasing fire intensity, driving enhanced land surface warming following larger fires.

Spatially, the summer ΔΤ following fire or harvest shows a clear warming effect when averaged over each 2º grid cell (Supplementary Figs. 16a–16b). The increasing relationship between summer ΔΤ and fire size, after controlling for F_dist_, largely persists spatially (Supplementary Fig. 16c, d), with β_ΔΤ, fire_ being significantly greater than β_ΔΤ, harvest_ (*p*<0.01, paired samples t-test, Supplementary Fig. 16e).

The hypothesis that the direct effect is due to surface roughness change was supported by a decreasing ratio of latent heat flux to surface net radiation as harvest patch size increases, when spatially explicit regressions are made for each 2º grid cell (Supplementary Fig. 17).

**Approach 2: manually maintaining a constant F_dist_ across patch size**

As an alternative approach, the greater value of β_ΔΤ, fire_ compared with β_ΔΤ, harvest_ was confirmed through manually maintaining a relatively unchanged F_dist_ when disturbance patch size increases, followed by the examination of the changes in summer ΔΤ with patch size. Through selecting appropriate F_dist_ thresholds for patches of different sizes for inclusion of 500-m pixels to calculate patch-level ΔΤ (in general, higher F_dist_ thresholds were used for patches with smaller sizes and vice versa, see Supplementary Table 4), we were able to maintain a constant F_dist_ (about 90%) across different patch sizes for both fire and harvest (Supplementary Figs. 18a, b), thus completely eliminating any co-varying F_dist_ effect with size. Again, ΔΤ was found to increase with size for both fire and harvest, with β_ΔΤ, fire_ being significantly (*p*<0.05, a multiple linear regression model with interaction terms between fire size and disturbance type was used) higher than β_ΔΤ, harvest_ (Supplementary Figs. 18c, d)_._

**Summary of both approaches**

In summary, the two approaches: (1) multiple linear regression (used to control for the effect of co-varying F_dist_ with patch size, Supplementary Table 3); (2) manually excluding any co-varying effect of F_dist_ (Supplementary Fig. 18), consistently indicate increasing ΔΤ with harvest patch size, providing robust evidence for a direct size effect in driving post-disturbance land surface warming. In addition, a consistently significantly higher β_ΔΤ, fire_ than β_ΔΤ, harvest_ was also found. The difference between β_ΔΤ, fire_ and β_ΔΤ, harvest_ ranges between 0.16 K [log_10_(km^2^)]^-1^ and 0.21 K [log_10_(km^2^)]^-1^, accounting for 31–33% of the derived values of β_ΔΤ, fire_, depending on the approach used. These results suggest the existence of additional surface warming from a covarying relationship between fire intensity and fire size, which contributes 31–33% of the observed enhanced surface warming with size.

**Evidence for the relationship between fire intensity and fire size**

The covarying relationship between fire intensity and fire size is supported by further analyses. The observed fire intensity, approximated by fire radiative power or FRP, was found to increase with the logarithm of fire size (Supplementary Fig. 19). Therefore, increasing fire intensity might also contribute to the observed increase in ΔΤ with fire size. A multiple linear regression, using ΔΤ as the dependent variable, with log_10_(size), FRP and F_dist_ as independent variables and using forest fire data only, shows that β_ΔΤ, fire_ decreased from 0.55 to 0.50 after FRP was additionally accounted for (Supplementary Table 5), indicating the contribution of FRP to the increasing relationship between ΔΤ and fire size. Note that β_ΔΤ, fire_ derived from the regression of ‘ΔΤ ~ log_10_(size) + F_dist_’ shown in Supplementary Table 5 is slightly different from that derived in Supplementary Table 3, because the samples for fire disturbance are different for these two cases, as ~50% of the fire patches have no associated FRP observations. For Supplementary Table 5, the sample size is 3728; for Supplementary Table 3, the sample size is 7509.

**Underlying biogeophysical processes**

The larger value of β_ΔΤ, fire_, compared with β_ΔΤ, harvest_, for post-disturbance ΔΤ in summer can be partly attributed to a lower surface albedo, which decreases with fire size, in contrast to a higher, and increasing, one with size for harvest (Supplementary Fig. 20, Supplementary Figs. 22, b). Surface warming in summer following forest harvest, despite an increase in surface albedo, is thus driven by a decreasing ecosystem evapotranspiration (ET) with harvest patch size (Supplementary Figs. 21d–f). For forest fire, the decreasing surface albedo with size in the following summer can be ascribed to increased surface charring, in line with increasing FRP with size. ET also decreased after fire (Supplementary Fig. 21a), but the decrease was generally less than after harvest (Supplementary Fig. 22c), with the slope between ΔET and log_10_(fire size) for fire also being lower than that for harvest (Supplementary Fig. 21d). Therefore, the decrease in surface albedo in summer after fire, which is mainly driven by increasing fire intensity, certainly contributed to the greater value of β_ΔΤ_ for fire than that for harvest.

**Supplementary Text 4 | Advantages of using fire size as the explanatory variable**

In addition to the ‘direct size effect’ in driving the increasing relationship between postfire surface warming and fire size as discussed in Supplementary Text 3, using fire size as the key explanatory variable has several advantages over using FRP or fire severity variables to explore surface climate impacts of forest fire and the implications of growing fire size on forest management, future climate change and fire activity.

- 1. **The dominance of direct size effect revealed by multiple linear regressions**

A multiple linear regression model incorporating log_10_(fire size), fire intensity (FRP), and fire severity (ΔLAI and postfire forest mortality) explained 47% of the variation in summer ΔΤ across the whole study domain of 40ºN–70ºN, which further confirmed the direct size effect in driving postfire summer surface warming after accounting for the co-varying fire behaviour variables (Supplementary Table 6). The same analysis was also performed for each 2º grid cell and confirms that the direct size effect also holds spatially (Supplementary Fig. 23a). Moreover, the partial coefficient of fire size was found to account for more than 50% of the β_ΔΤ_ values derived from simple regressions (Supplementary Table 6, Supplementary Figs. 23b, c), suggesting that the direct size effect best explains postfire summer surface warming, which justifies using fire size as the key explanatory variable to investigate postfire biogeophysical changes.

- 1. **Uncertainties and availability of FRP data**

Fire Radiative Power (FRP) measures the rate of radiative energy emitted by an active fire within a given satellite pixel observed at the moment of satellite overpass and only if the observations are not obstructed by clouds. Although it has been demonstrated that active fire detection can succeed even the thick smoke^7^ resulting from active burning, pixels with thick smoke are often inadvertently masked out by conservative cloud masking, hence reducing the chance that actively burning fires are detected under heavy smoke^8^.

While FRP data are the only direct observations available at the global scale to be used as a proxy for fire intensity, important challenges are associated with the analysis and interpretation of this data source. Large-scale FRP observations are only available for the era of satellite observation, with the current reliable and longest time series being MODIS data which are only available for the period since 2001. Given the large interannual variations of forest fires, this limited data availability diminishes their utility for the analysis of any long-term trends in fire intensity.

More fundamentally, FRP is an imperfect proxy for fire intensity because it has a low sensitivity to the fraction of the ground being burned within the satellite pixel and because it integrates the radiative energy emitted by all forms of combustion occurring: the active fire front, as well as smoldering stems, the organic layer, peat etc., after the passing of fire front. The fact that FRP is insensitive to the fraction of the pixel being actively burned can result in the same FRP value being obtained for small fire fronts with a high frontline intensity and for larger fire fronts with a low frontline intensity. Hence, MODIS data show similar FRP values for Canadian boreal forest fires, central Asian grassland fires, and tropical savanna fires (Fig. 1 in Wooster et al.^8^), which have strikingly different fire regimes and frontline fire intensities. Furthermore, pixels behind the active fire line which are smoldering still give a (low) FRP value, but these cannot be separated from values from the active fire line. Therefore, typically, a very wide range of FRP values are observed within a single fire, complicating the analysis.

Last, due to the limitations in FRP observation mentioned above, FRP observations are more scarce than burned area and fire size observations. Over our study domain of 40ºN–70ºN, 41% of fire patches have no associated FRP observations, further limiting the scope of potential data analysis. In addition, a recent review on satellite remote sensing of active fires^8^ pointed out that the current validation level of satellite FRP products is less advanced than that for burned area, partly due to the ephemeral nature of active fires and the logistical and technical difficulties posed when trying to get independent, simultaneous FRP observations to match satellite estimates. Hence, burned area observations, and the associated fire size data (the uncertainties in fire size data linked to the definition of fire size does not compromise our conclusion, detailed below), are likely more accurate than FRP observations.

- 1. **Limitations of forest mortality or fire-induced LAI change**

The fire severity data used in this study, including postfire forest mortality and LAI change, need additional and dedicated data processing compared to fire size data. Unlike fire size or FRP, satellite-derived observations of forest mortality or LAI change following fire are not readily available. Neither are we aware of any major countries or forest agencies that routinely measure and maintain datasets of forest mortality and/or LAI change after fire.

In addition, partial stand mortality remains difficult to detect using satellite observations and would need extensive field observations for algorithm calibration. Hence, in this study, forest mortality consists only of mortality events detected as stand-replacing ones. LAI change following fire is subject to both the effects of climate variations and disturbances including fire and potentially other forms of disturbance (i.e., drought, wind, insect, etc.). Hence, as in this study, the determination of fire-induced LAI change requires the removal of the effects of climate variations and disturbances other than fire.

In contrast, fire size information is routinely maintained by major countries subject to frequent wildland forest fires, including USA, Canada, Australia and European countries. Of course, fire size datasets also contain uncertainties associated with burned pixel detection and estimates of the date of burning and the definition of a fire patch. For example, a fire patch can be defined from the point view of fire propagation (i.e., burned pixels comprising a fire patch need to be spatially connected and temporally consecutive, e.g., Global Fire Atlas) or simply by grouping burned pixels into fire patches as long as the burned pixels are spatially connected (as in Supplementary Text 3). However, our study shows that the reported increasing relationship between postfire surface warming and fire size holds for datasets built following either of these two definitioins. Moreover, given that burned area mapping is under continual improvement by the remote sensing community (e.g., the recent availability of burned area at a fine resolution of 20m from the Sentinel sensor^9^), the mapping of fire size is expected to continue to improve and its associated uncertainties are likely to be further reduced.

- 1. **Implications of fire size for forest management**

Fire size has more direct implications for forest fire management than FRP, forest mortality or LAI change. The propagation of a fire, and thereby its final size, can be actively curbed or stopped given adequate resources, but it would be difficult to use FRP, forest mortality or LAI change as a direct fire management target. At the local scale, it is possible that FRP or fire severity (forest mortality or LAI change) can quickly saturate although the burning can continue and the fire patch can continue to expand. For instance, if fire weather reaches its extreme state with critically low fuel moisture at the very start of burning, then intensity can quickly reach its maximum and saturate, but fire size will continue to grow if fire propagation is not curbed. In this case, the ‘direct size effect’, i.e., the effect of surface roughness associated with the spatial extent of burning will continue to grow and affect land surface temperature, as long as the fire patch continues to expand.

As FRP, forest mortality and postfire LAI change largely correlate with fire size, these effects could be accounted for in discussions of the biogeophysical impacts of fire size. But fire size is unique in that many ecological processes depend on the spatial scale of a burned patch. For example, the size of a burned patch influences the dispersal distance that plant propagules need to travel for seed germination and seedling recruitment, and therefore influences postfire stand regeneration. This process, although occurs mainly within the first few years after fire, has an impact lasting for decades on postfire vegetation succession, associated ecosystem service (including carbon cycle and climate regulation), and local fire cycle. Fire size can also influence the distance that an animal needs to travel for feeding and the subsequent exposure to potential predators. For ecosystem management, such ecological effects associated with fire size cannot be easily related to FRP, forest mortality or LAI change.

Supplementary Text 5 | The tiling effect

The Global Fire Atlas (GFA)^10^ cuts single fires which cross two MODIS tiles into two individual fires and hence can underestimate fire size. Because each MODIS tile covers a domain of 10º by 10º (i.e., about an area of 600,000 km^2^ at a latitude of 60ºN), we expect that most fire patches would completely fall within the borders of an individual tile. Our analysis, using the 2016 fire season as an example, confirmed this expectation: the fire patches whose perimeters were in contact with MODIS tile borders accounted for 0.63% of the total number of fire events (n=10952), with their burned area accounting for 3.74% of the total burned area. These percentages represent the upper boundary of the importance of fire patches subject to the tiling issue in GFA for the 2016 fire season. It is, therefore, hypothesized that the GFA tiling effect has only a limited influence on our main conclusions.

To test this hypothesis, we used GlobFire^11^, another fire patch dataset which is based on the same MCD61A1 burned area data underlying GFA but has no known tiling issues. A spatial pattern of postfire land surface warming (for the summer one year after fire) increasing with fire size, similar to that shown in main text Fig. 1c (based on GFA), was derived using GlobFire (Supplementary Fig. 24a). This confirms the hypothesis that the tiling issue in GFA does not influence the robustness of our conclusion that fire size amplifies postfire surface warming. Nonetheless, the slope between postfire surface warming and fire size (β_ΔΤ_) derived using GlobFire seems lower than that derived using GFA (Supplementary Fig. 24b), probably because GlobFire tended to report more small fires compared to GFA (Supplementary Figs. 25a, b), although both fire patch datasets were based on the MCD61A1 burned area data.

The fact that GlobFire reports more small fires compared to GFA does not come as a complete surprise because different algorithms and parameters were used to reconstruct fire patches from the underlying MODIS burned pixels in the two products. More specifically, GlobFire had a higher frequency of fires ranging from 10^0^ to 10^0.4^ km^2^ than GFA and a lower frequency of fires ranging from 10^0.5^ to 10^2^ km^2^ (Supplementary Fig. 25a). Correspondingly, the summer ΔT derived from GlobFire was higher than that derived from GFA for fires ranging from 10^0^ to 10^0.4^ km^2^, and in contrast, lower than that from GFA for fires ranging from 10^0.5^ to 10^2^ km^2^ (Supplementary Fig. 25c). This observation is compatible with the underlying increasing relationship between ΔΤ and fire size, because GlobFire likely divided large fires with a high ΔT erroneously into several small ones: ΔT for small fires was inflated in GlobFire, whereas the reverse is true for large fires. This further explains the lower value of β_ΔT_ derived from GlobFire compared with the value from GFA.

In conclusion, the amplifying effect of fire size in postfire land surface warming derived by using GFA was consistent with that from GlobFire, demonstrating that the tiling effect in GFA is too small to alter the main findings of this study.

Supplementary Text 6 | The edge effect

Pixels at the edge of a given fire patch could potentially have lower magnitudes of surface warming than those in the interior either because of potentially less complete burning or because land surface energy processes driving land surface temperature (LST) change in these edge pixels were buffered by neighboring unburned pixels. As fire size increases, the fraction of the area occupied by edge pixels will decrease, driving a potentially increasing mean value of ΔLST of the entire patch (i.e., the so-called edge effect). Here, we test whether the main findings of the increasing relationship between ΔLST and fire size could be due to the artifact of the edge effect.

Consider a simple theoretical model where a fire patch is represented by a circular area consisting of an interior circle with a radius *r* and an edge with width *d* (Supplementary Fig. 26). Suppose that the postfire surface temperature change is ΔT_i_ for the interior and ΔT_e_ for the edge, then the temperature change of the entire patch (ΔT) can be written as:

$\Delta T=\frac{\pi r^{2}\Delta T_{i}+2\pi rd\Delta T_{e}+\pi d^{2}\Delta T_{e}}{\pi\left(r+d\right)^{2}}$ Eq. (S1)

As explained above, the edge effect assumes that the LST change in the interior is greater than that at the edges (i.e., ΔT_i_ > ΔT_e_). It is clear from Equation (S1) that ΔT for the entire patch, if driven by the so-called ‘edge effect’, will saturate and will approach ΔT_i_ as an asymptote when fire size becomes sufficiently large. This is different from our observations, in which ΔT shows no sign of saturation with increasing fire size (Supplementary Figs. 10b–10f; Supplementary Figs. 15a, b). The lack of saturation can be explained if ΔT_i_ also becomes larger as *r* increases, which provides an independent line of evidence confirming our main finding, i.e., postfire surface warming increases with fire size.

As an empirical example, changes in ΔT_i_, ΔT_e_ and ΔT in summer were calculated for different fire sizes in US Alaska and Canada for 2003–2006 using the Global Fire Atlas dataset (Supplementary Fig. 27). Here *d* was chosen as 500m (i.e., a single MODIS pixel). For small fires, it is not necessary that ΔΤ_i_ is larger than ΔT_e_ because of the variations in ΔΤ (Supplementary Fig. 27c). However, when being averaged within different bins of fire size, ΔΤ_i_ is generally greater than ΔT_e_ for big fires (Supplementary Fig. 27e). In addition, both ΔT_i_ and ΔT_e_ increased with fire size (Supplementary Figs. 27a, b). The observation that ΔT_i_ (thus, excluding the edge effect) increased with fire size (β=0.64 K [log_10_(km^2^)]^-1^, *p*<0.05), demonstrates that the reported amplification effect of fire size is not an artifact of the edge effect. Moreover, the positive relationship between ΔT_e_ and fire size, albeit with a low β value (0.36 K [log_10_(km^2^)]^-1^, *p*<0.05), indicates that even ΔΤ at the edges was possibly influenced by elevated fire intensity with increasing fire size. The difference between ΔΤ_i_ and ΔΤ_e_ also significantly increased with fire size (Supplementary Fig. 27c), probably partly because of a greater increase in fire intensity within the interior of a fire patch than at its edge, which is buffered by unburned areas. Finally, ΔT of the entire fire patch increased with fire size but the β value is more driven by the warming in the interior region (Supplementary Figs. 27d, e).

In conclusion, the ‘edge’ effect can contribute to the uncertainty in β_ΔΤ_ but the increasing relationship between postfire ΔΤ and fire size is not an artifact of the edge effect.

**References**

1 White, J. C., Wulder, M. A., Hermosilla, T., Coops, N. C. & Hobart, G. W. A nationwide annual characterization of 25 years of forest disturbance and recovery for Canada using Landsat time series. *Remote Sens. Environ.* **194**, 303-321 (2017).

2 Alkama, R. & Cescatti, A. Biophysical climate impacts of recent changes in global forest cover. *Science* **351**, 600-604 (2016).

3 Li, Y. *et al.* Local cooling and warming effects of forests based on satellite observations. *Nat. Commun.* **6**, 6603 (2015).

4 Duveiller, G., Hooker, J. & Cescatti, A. The mark of vegetation change on Earth's surface energy balance. *Nat. Commun.* **9**, 679 (2018).

5 Liu, Z., Ballantyne, A. P. & Cooper, L. A. Biophysical feedback of global forest fires on surface temperature. *Nat. Commun.* **10**, 214 (2019).

6 Forzieri, G., Alkama, R., Miralles, D. G. & Cescatti, A. Satellites reveal contrasting responses of regional climate to the widespread greening of Earth. *Science* **356**, 1180-1184 (2017).

7 Atwood, E. C. *et al.* Detection and Characterization of Low Temperature Peat Fires during the 2015 Fire Catastrophe in Indonesia Using a New High-Sensitivity Fire Monitoring Satellite Sensor (FireBird). *PLoS One* **11**, e0159410 (2016).

8 Wooster, M. J. *et al.* Satellite remote sensing of active fires: History and current status, applications and future requirements. *Remote Sens. Environ.* **267**,112694 (2021).

9 Roteta, E., Bastarrika, A., Padilla, M., Storm, T. & Chuvieco, E. Development of a Sentinel-2 burned area algorithm: Generation of a small fire database for sub-Saharan Africa. *Remote Sens. Environ.* **222**, 1-17 (2019).

10 Andela, N. *et al.* The Global Fire Atlas of individual fire size, duration, speed and direction. *Earth Syst. Sci. Data* **11**, 529-552 (2019).

11 Artes, T. *et al.* A global wildfire dataset for the analysis of fire regimes and fire behaviour. *Sci. Data* **6**, 296 (2019).

**Supplementary figures**


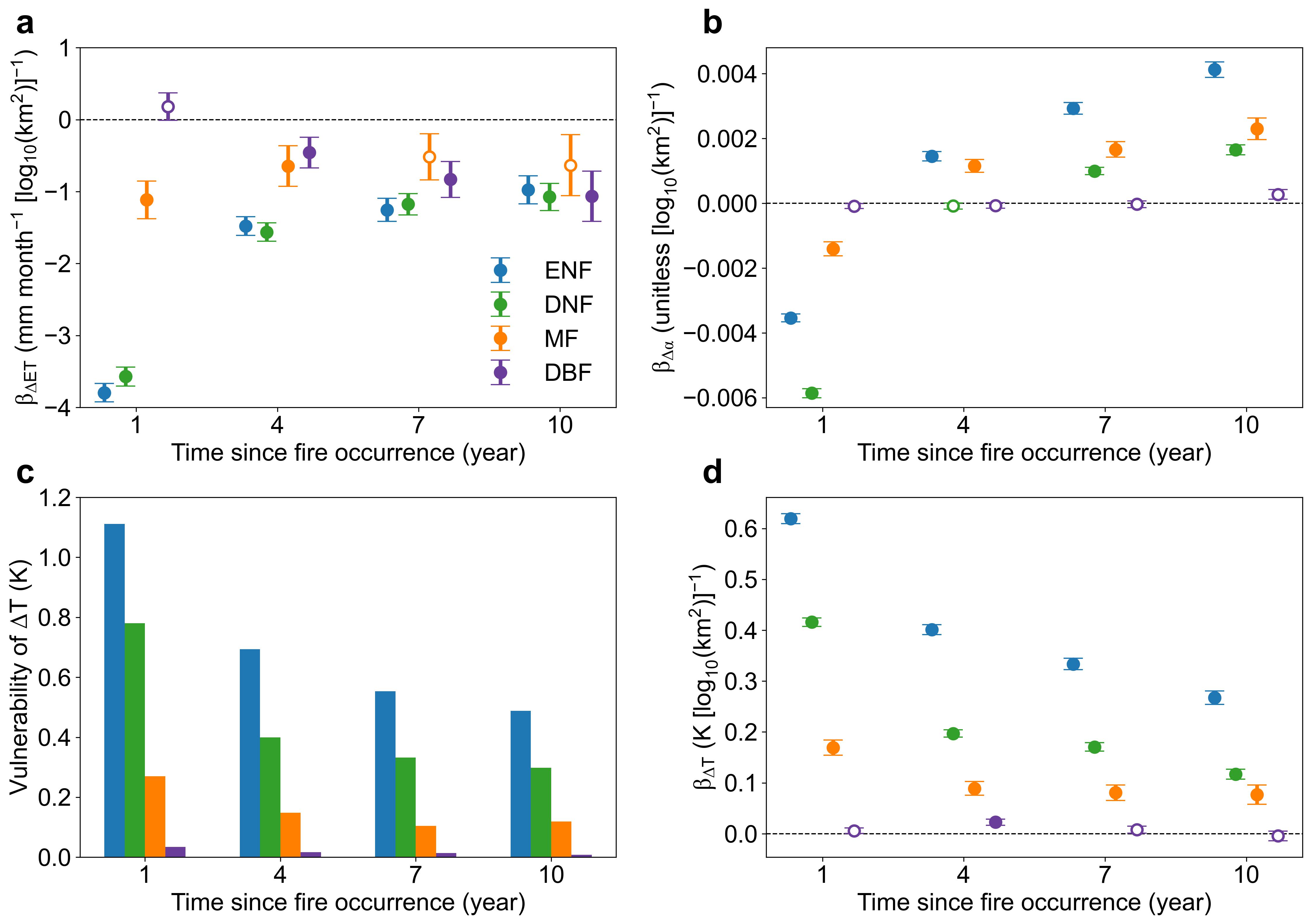


**Supplementary Figure 1 | Postfire biogeophysical changes amplified by fire size for up to 10 years after fire, stratified by forest type.** Simple linear regression models (y = α + β × log_10_(fire size)) were fitted to derive the effect of fire size (β) on postfire biogeophysical changes in summer (June–August) for each forest type, where y stands for postfire changes in evapotranspiration (β_ΔET_, **a**), surface albedo (β_Δα_, **b**) and surface radiometric temperature (β_ΔT_, **d**). Panel (**c**) shows fire vulnerability in terms of postfire changes in summer land surface temperature (ΔΤ), defined as the average ΔΤ over all fires of a given forest type. Values for one year, four years, seven years, and ten years after fires are shown. Solid (empty) dots represent statistically significant (insignificant) β values (*p*<0.05, the student’s t-test). Error bars indicate standard errors. Figure developed using the Python open-source tools.





**Supplementary Figure 2 | Fire size impact on surface radiometric temperature change (ΔΤ) one year after fire in forests of the continental USA.** The Global Fire Atlas dataset, covering the years 2003 to 2016, was used. The first column of panels (**a**, **d**, **g**) displays the mean ΔΤ for each 2º grid cell. The second (**b**, **e**, **h**) and third (**c**, **f**, **i**) columns show the regression slope (β_ΔΤ_) and the coefficient of determination (R^2^), derived from fitting a linear regression model (ΔΤ = α + β_ΔΤ_ × log_10_(fire size)) for each 2º grid cell, including all fire patches larger than 1km^2^ within a grid cell containing more than 10 fires. Both solid and empty dots indicate pixels with locally significant regressions (*p*<0.05, the two-tailed t-test), but solid dots indicate those having passed a more rigorous field significance test corrected for the false discovery rate (α_FDR_ = 0.10, see Methods). The first row of panels shows the annual time scale; the second row shows summer (June–August); the third row shows winter (December–February). Figure developed using the Python open-source tools.





**Supplementary Figure 3 | Fire size impact on surface radiometric temperature change (ΔΤ) one year after fire in Australian forests.** The Global Fire Atlas dataset, covering the years 2003 to 2016, was used. The first column of panels (**a**, **d**, **g**) displays the mean ΔΤ for each 2º grid cell. The second (**b**, **e**, **h**) and third (**c**, **f**, **i**) columns show the regression slope (β_ΔΤ_) and the coefficient of determination (R^2^), derived from fitting a linear regression model (ΔΤ = α + β_ΔΤ_ × log_10_(fire size)) for each 2º grid cell, including all fire patches larger than 1km^2^ within a grid cell containing more than 10 fires. Both solid and empty dots indicate pixels with locally significant regressions (*p*<0.05, the two-tailed t-test), but solid dots indicate those having passed a more rigorous field significance test corrected for the false discovery rate (α_FDR_ = 0.10, see Methods). The first row of panels is for the annual time scale; the second row for summer (December–February); the third row for winter (June–August). Figure developed using the Python open-source tools.


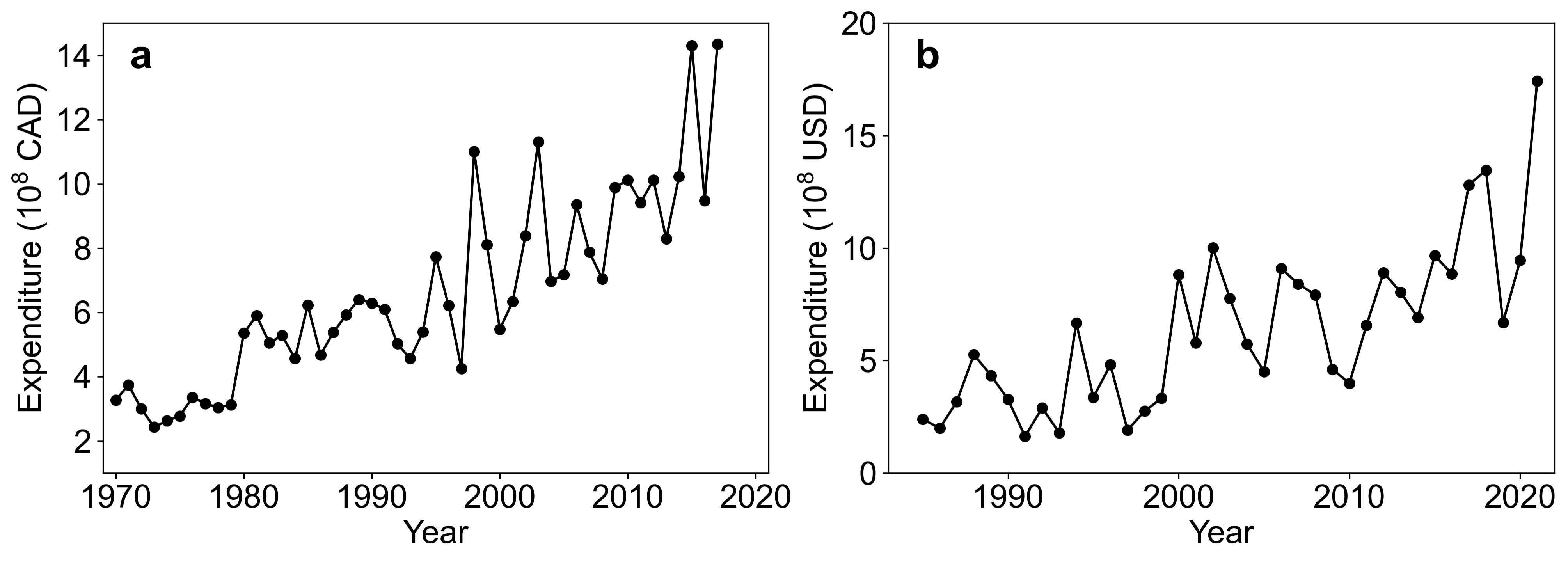


**Supplementary Figure 4 | Temporal changes in expenditure on forest fire control for Canada (a) and the USA (b).** Influences of inflation in currency were accounted for in both datasets. The expenditure in Canada (based on the 2017 value of the Canadian dollar; CAD) refers to the “cost of wildland fire protection” and consists of expenses for fire preparedness, mitigation, response, and recovery. The expenditure in the USA (based on the 1985 value of the U.S. dollar or USD) refers to the “Federal Firefighting Cost” and includes only the cost of fire suppression. See Supplementary Table 7 for information on the data sources. Figure developed using the Python open-source tools.

**
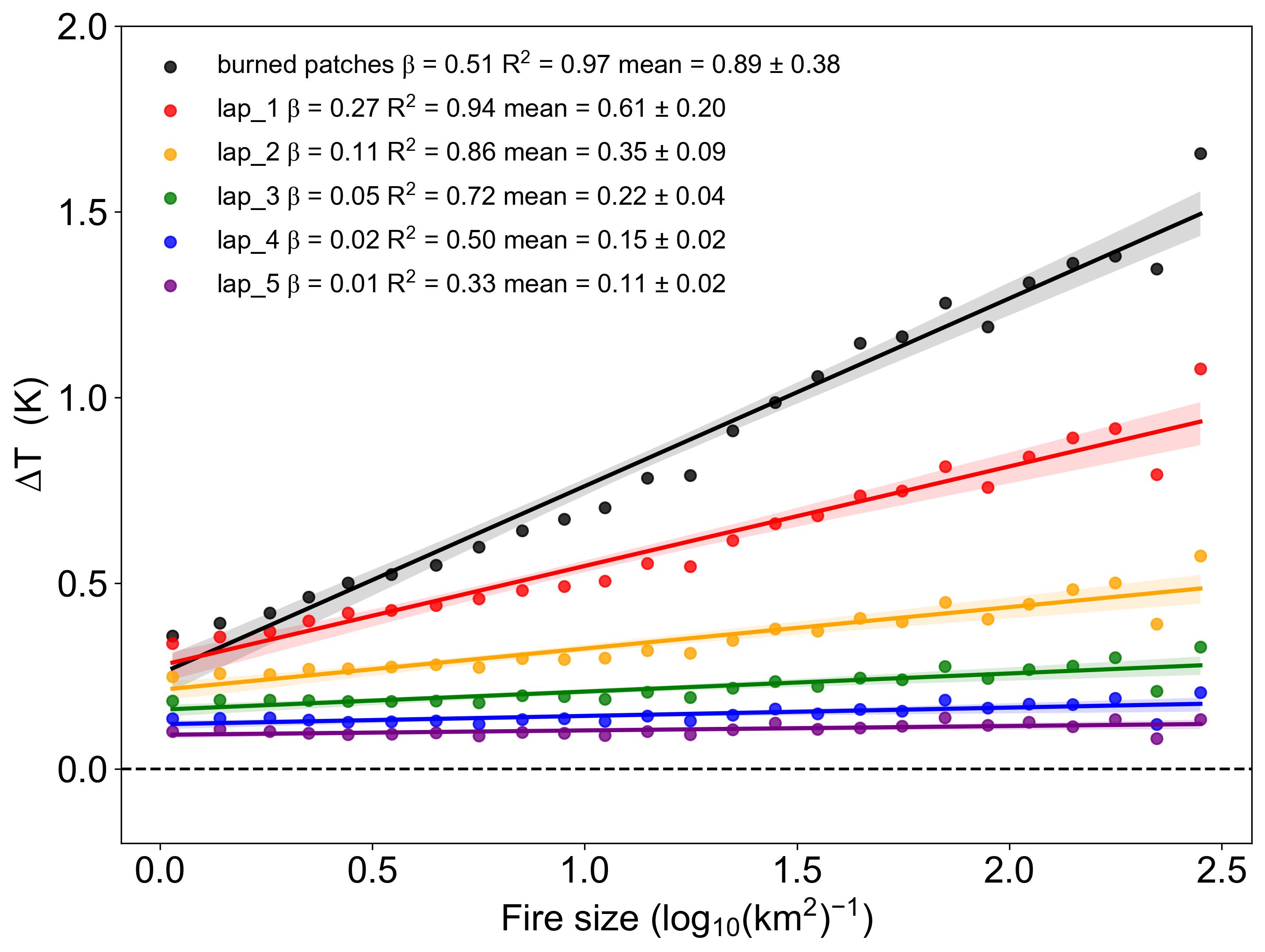
**

**Supplementary Figure 5 | Peripheral unburned forests outside burned perimeters show land surface warming amplified by fire size.** Changes in land surface temperature (ΔΤ) in summer (June–August), one year after fire, were examined over peripheral unburned forests outside burned perimeters using the same ‘space-and-time’ approach used to derive ΔΤ for burned patches (see Section 2.1 in Methods), but with the candidate pixels used to derive ΔΤ_res_ being located outside all peripheral areas. A distance of up to 5 MODIS pixels (roughly equivalent to 2.5 km), with each circle having a width of one MODIS pixel (roughly equivalent to 500m), was examined until the slope between ΔΤ and fire size became negligible. The linear relationships between ΔΤ and fire size are shown for burned patches (black), and for unburned forests with distances from burned perimeters of 500m (‘lap_1’, red), 500-1000m (‘lap_2’, orange), 1000-1500m (‘lap_3’, green), 1500-2000m (‘lap_4’, blue), and 2000-2500m (‘lap_5’, purple), respectively. Fire events and the associated peripheral circles of different distances were grouped according to the size of burned patches, with intervals of 0.1 in the logarithmic scale. The mean value of ΔT were derived for each bin. β and R^2^ represent the linear regression slope and the coefficient of determination. The mean values of ΔΤ and the standard deviations are also shown. Shading denotes 95% prediction intervals. All regressions are significant (n=25, *p*<0.05, the student’s t-test). A linear regression model (ΔΤ = α + β × log_10_(fire size)) was also performed using original data without binning, with β values for burned patches, lap_1, lap_2, lap_3, lap_4 and lap_5 being 0.44 (*p*<0.01, R²=0.076), 0.23 (*p*<0.01, R²=0.030), 0.08 (*p*<0.01, R²=0.007), 0.03 (*p*<0.01, R²=0.001), 0.01 (*p*<0.01, R²=0.001), and 0.01 (*p*<0.01, R²=0.001), respectively. Figure developed using the Python open-source tools.


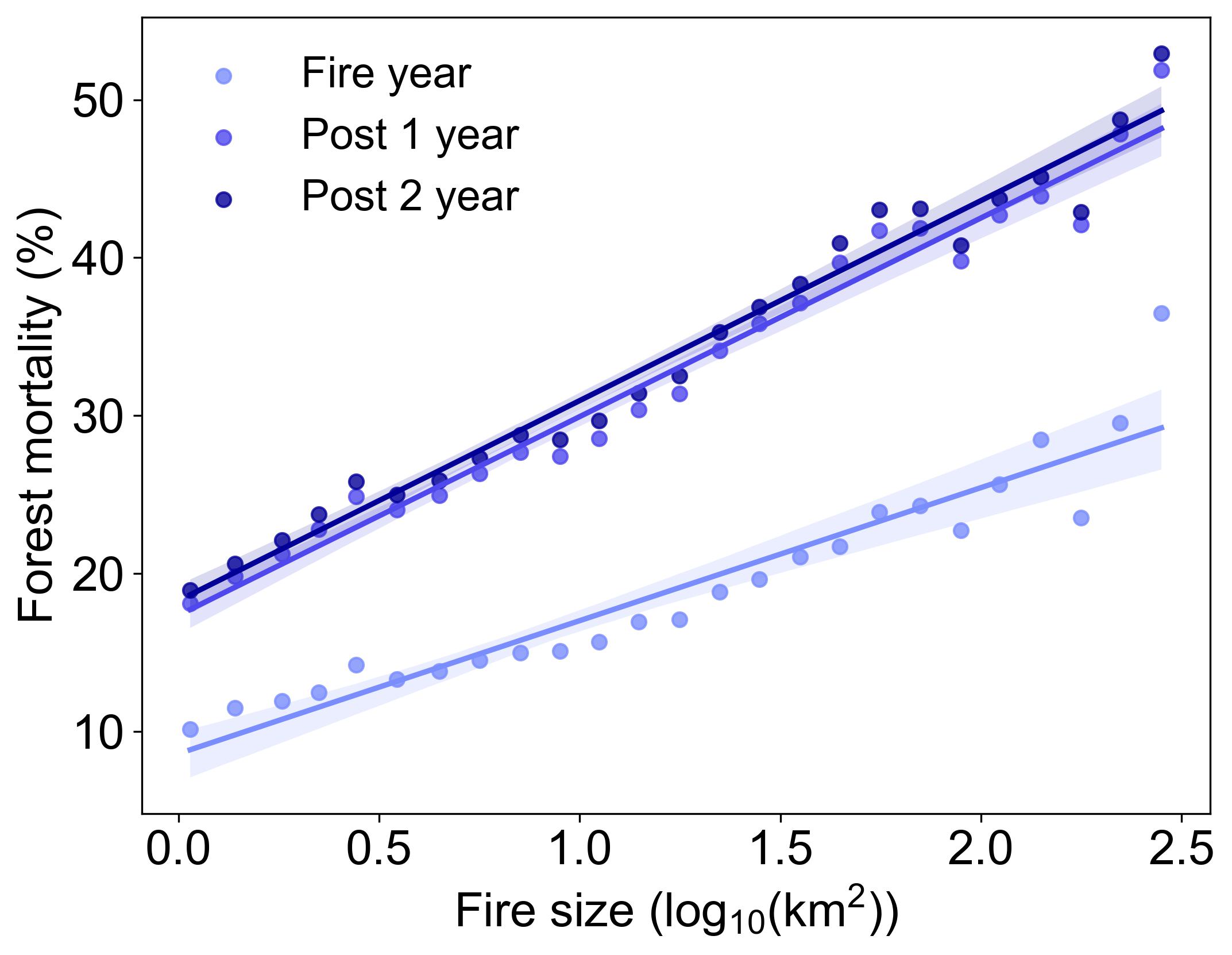


**Supplementary Figure 6 | Linear relationships between fire-induced forest mortality and fire size obtained by using different time windows.** Forest mortality rates were calculated by including forest deaths within the year of fire (i.e., within the year of burning, in light blue, β=8.4% [log_10_(km^2^)]^-1^, *p*<0.01, R^2^=0.90), 1 year (2-year time window, in blue, β=12.5% [log_10_(km^2^)]^-1^, *p*<0.01, R^2^=0.97) and 2 years (3-year time window, in dark blue, β=12.6% [log_10_(km^2^)]^-1^, *p*<0.01, R^2^=0.97) after fire. Fire events were grouped into different bins with intervals of 0.1 in the logarithmic scale, with the mean forest mortality being derived for each bin. β and R^2^ represent the linear regression slope and the coefficient of determination, respectively (n=25, with the significance test being made using the student’s t-test). Shading denotes 95% prediction intervals. A linear regression model (mortality = α + β × log_10_(fire size)) was also performed using original fire patch data without binning, with β values for the year of the fire, 1 year after the fire, and 2 years after the fire being 6.6% [log_10_(km^2^)]^-1^ (*p*<0.01, R²=0.02), 11.6% [log_10_(km^2^)]^-1^ (*p*<0.01, R²=0.04), and 11.7% [log_10_(km^2^)]^-1^ (*p*<0.01, R²=0.04), respectively. Figure developed using the Python open-source tools.





**Supplementary Figure 7 | Fire size impact on fire-induced forest mortality** **across northern forests (40ºN–70ºN) obtained using different time windows.** The first (**a–c**), second (**d–f**) and third (**g–i**) rows show the results derived using the time window of 0-year (i.e., including forest loss within the year of burning), 1-year (including forest loss for up to 1 year after burning) and 2–year (including forest loss for up to 2 years after burning), respectively. The first column of panels displays the mean mortality for each 2º grid cell. The second and third columns show the regression slope (β) and the coefficient of determination (R^2^), respectively, derived by fitting a linear regression model (mortality = α + β × log_10_(fire size)) within 2º grid cells containing more than 10 fires. Both solid and empty dots indicate pixels with locally significant regressions (*p*<0.05, the two-tailed t-test), but solid dots indicate those having passed a more rigorous field significance test corrected for the false discovery rate (α_FDR_ = 0.10, see Methods). The light grey background in all maps indicates northern forests with a >10% ground coverage. Figure developed using the Python open-source tools.





**Supplementary Figure 8 | Fire size impact on postfire summer (June–August) land surface temperature change (ΔΤ) derived using different window sizes in the “time and space” method** **across northern forests (40ºN–70ºN).** The first (**a–c**), second (**d–f**) and third (**g–i**) rows represent the results obtained based on window sizes of 50km×25km, 25km×25km, and 50km×50km, respectively. The first column of panels displays the mean ΔΤ for each 2º grid cell. The second and third columns show the regression slope (β_ΔΤ_) and the coefficient of determination (R^2^), respectively, derived by fitting a linear regression model (ΔΤ = α + β_ΔΤ_ × log_10_(fire size)) within 2º grid cells containing more than 10 fires. Solid dots indicate pixels with locally significant regressions (*p*<0.05, the two-tailed t-test). All significant regressions have passed a more rigorous field significance test corrected for the false discovery rate (α_FDR_ = 0.10, see Methods). The light grey background in all maps indicates northern forests with a >10% ground coverage. Figure developed using the Python open-source tools.


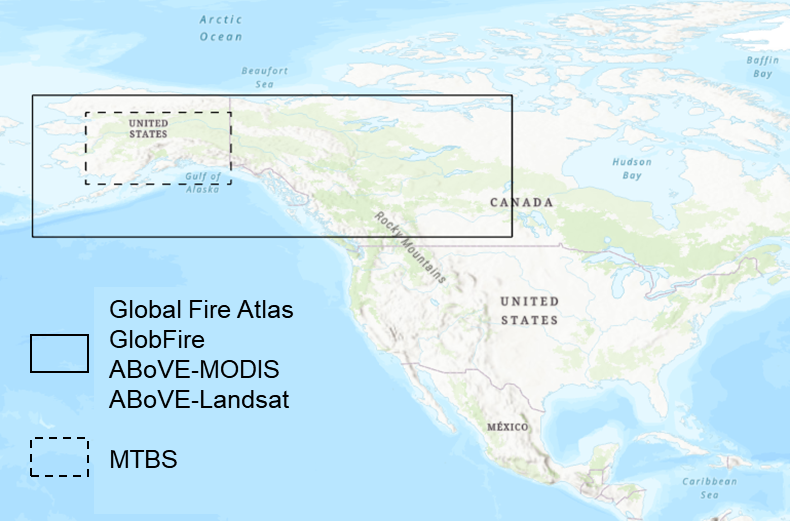


**Supplementary Figure 9 | The spatial domains of different fire patch datasets.** All fire patch datasets covered the same region of North American boreal forests in US Alaska and western and central Canada except for the MTBS data which covered only Alaska.


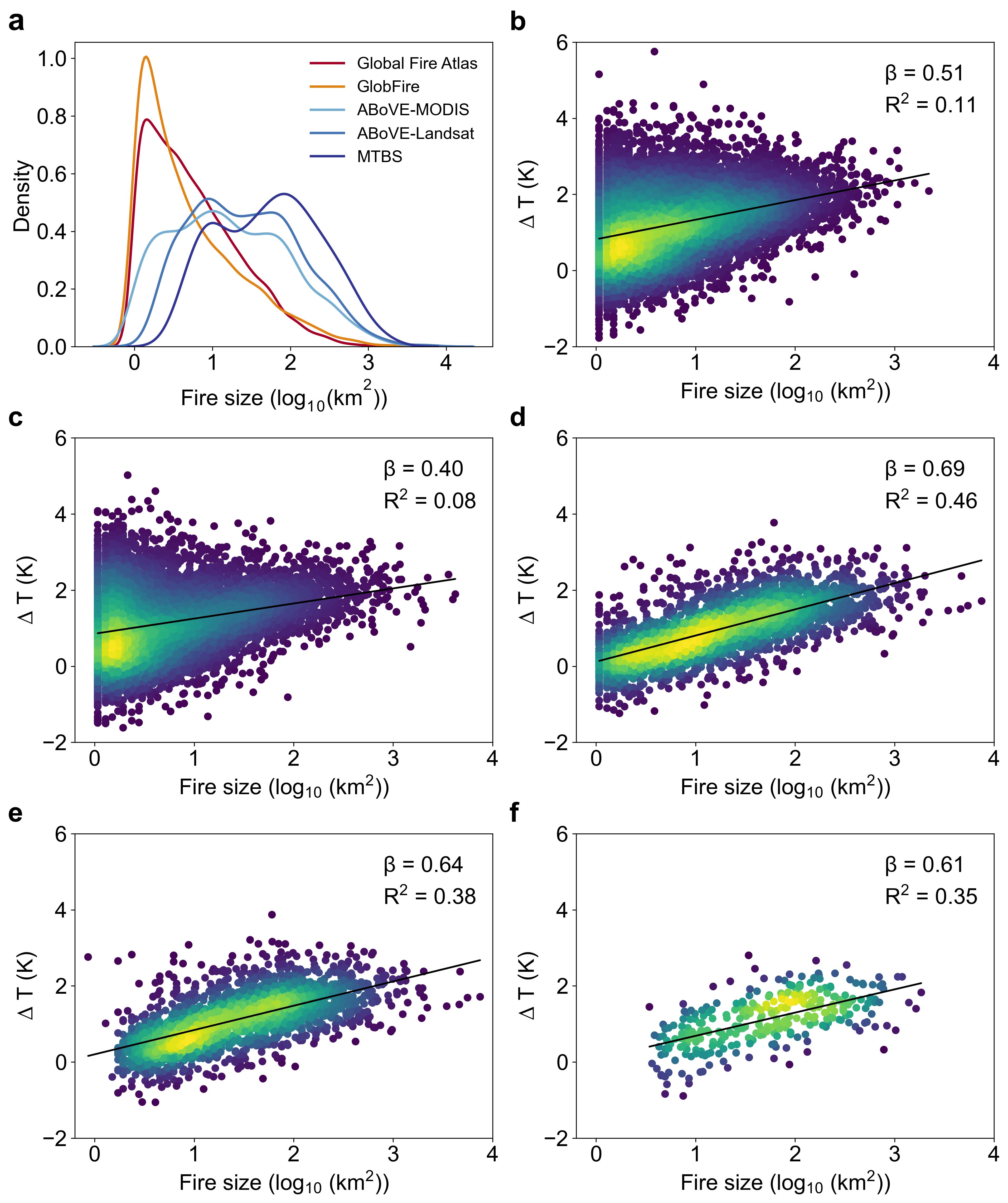


**Supplementary Figure 10 | The distribution probability density curves of fire size (a) and the relationships between postfire changes in summer (June–August) land surface temperature (ΔΤ) and fire size (b-f) derived from different fire patch datasets.** Panels (**b**–**f)** represent the results from the Global Fire Atlas, GlobFire, ABOVE-MODIS, ABOVE-Landsat, and MTBS, respectively. The analysis covers the period 2003–2015, during which all datasets were available. Linear regressions (ΔΤ = α + β_ΔΤ_ × log_10_(fire size)) were fitted between ΔΤ and fire size, with all regressions being significant (*p*<0.05, the student’s t-test). β and R^2^ represent the regression slope and the coefficient of determination, respectively. Figure developed using the Python open-source tools.





**Supplementary Figure 11 | Postfire surface warming in summer (June–August) and its amplification by fire size derived from different fire patch datasets at a 2º resolution.** Panels (**a**–**c**), (**d**–**f**), (**g**–**i**), (**j**–**l**) and (**m**–**o**) represent the results from the Global Fire Atlas, GlobFire, ABOVE-MODIS, ABOVE-Landsat, and MTBS, respectively. The analysis covered the period 2003–2015, during which all datasets were available. The first column of panels displays the mean ΔΤ for each 2º grid cell. The second and third columns show the regression slope (β_ΔΤ_) and the coefficient of determination (R^2^), respectively, derived by fitting a linear regression model (ΔΤ = α + β_ΔΤ_ × log_10_(fire size)) within the 2º grid cells containing more than 10 fires. Solid dots indicate pixels with locally significant regressions (*p*<0.05, the two-tailed t-test). All significant regressions have passed a more rigorous field significance test corrected for the false discovery rate (α_FDR_ = 0.10, see Methods). Figure developed using the Python open-source tools.


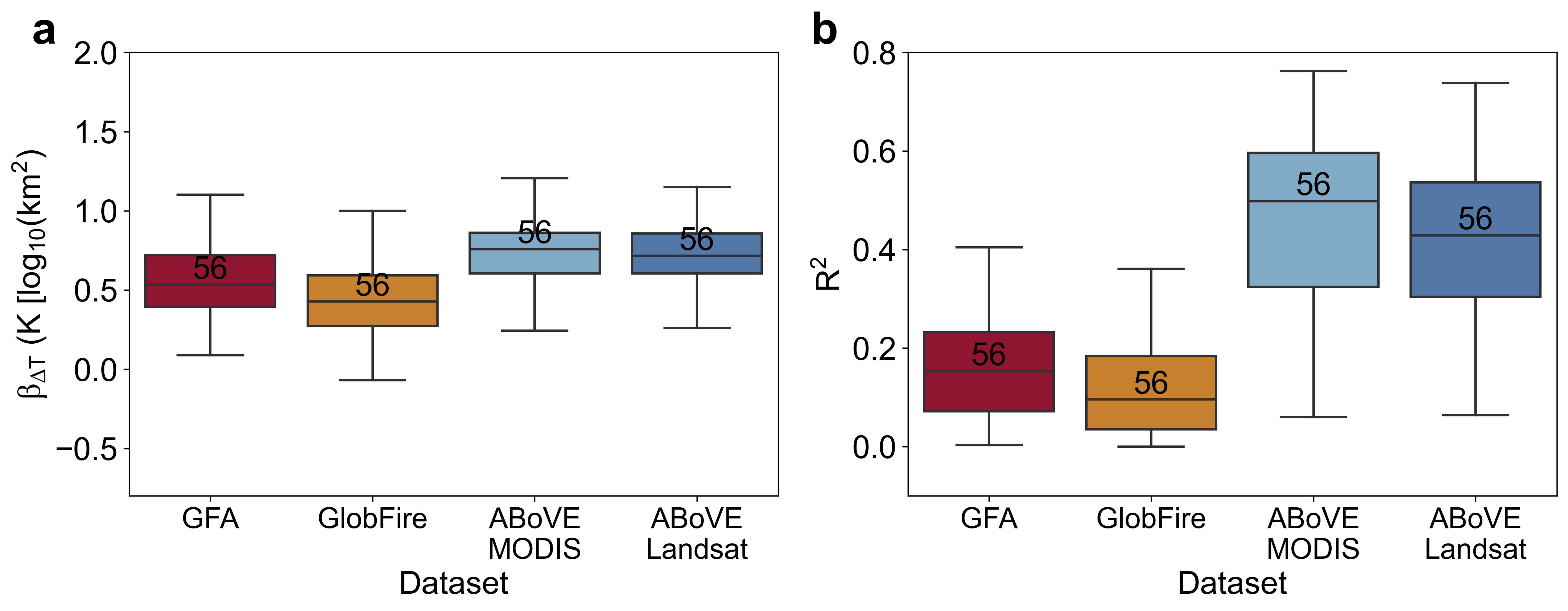


**Supplementary Figure 12 | Comparison of the amplification effect of fire size on the change in summer (June–August) surface radiometric temperature (ΔT) one year after fire according to different fire patch datasets.** Simple linear regression models (ΔT = α + β_ΔT_ × log_10_(fire size)) were fitted to derive the amplification effect (β_ΔT_) of fire size on postfire changes in ΔT in summer one year after fire for each 2º grid cell (shown in Supplementary Fig. 11). Panels (**a**) and (**b**) show the statistical distribution of βΔT (the regression slope) and R^2^ (the coefficient of determination), respectively. The spatial domain of 2º grid cells used to derive the boxplots cover the overlapping areas (n=56) of the four datasets, Global Fire Atlas (GFA), GlobFire, ABOVE-MOIDS and ABOVE-Landsat. The center line of the boxplots represents the median value, with box limits indicating upper and lower quartiles and whiskers showing 1.5 × interquartile range. Figure developed using the Python open-source tools.

**

**

**Supplementary Figure 13 | Biogeophysical and surface energy flux changes in winter (December–February) one year after fire and the effects of fire size over northern forests (40ºN–70ºN).** The first and third columns show the mean values of postfire changes in land surface temperature (ΔT, **a**), outgoing longwave radiation (ΔLW_out_, **c**), surface albedo (Δα, **e**), reflected shortwave radiation (ΔSW_out_, **g**), ecosystem evapotranspiration (ΔET, **i**), latent heat flux (ΔLE, **k**), the sum of sensible and ground heat fluxes (Δ(H+G), **m**) and net radiation (ΔR_n_, **n**) by averaging all fire within each 2º grid cell. The second and fourth columns show linear regression slopes (β) derived by fitting a linear regression model (y = α + β × log_10_(fire size)) for each 2º grid cell with more than 10 fires. In the regression model, y stands for ΔT (**b**), ΔLW_out_ (**d**), Δα (**f**), ΔSW_out_ (**h**), ΔET (**j**), ΔLE (**l**), Δ(H+G) (**n**), and ΔR_n_ (**p**). Both solid and empty dots indicate pixels with locally significant regressions (*p*<0.05, the two-tailed t-test), but solid dots indicate those having passed a more rigorous field significance test corrected for the false discovery rate (α_FDR_ = 0.10, see Methods). The light grey background in all maps indicates northern forests with a >10% ground coverage. Figure developed using the Python open-source tools.


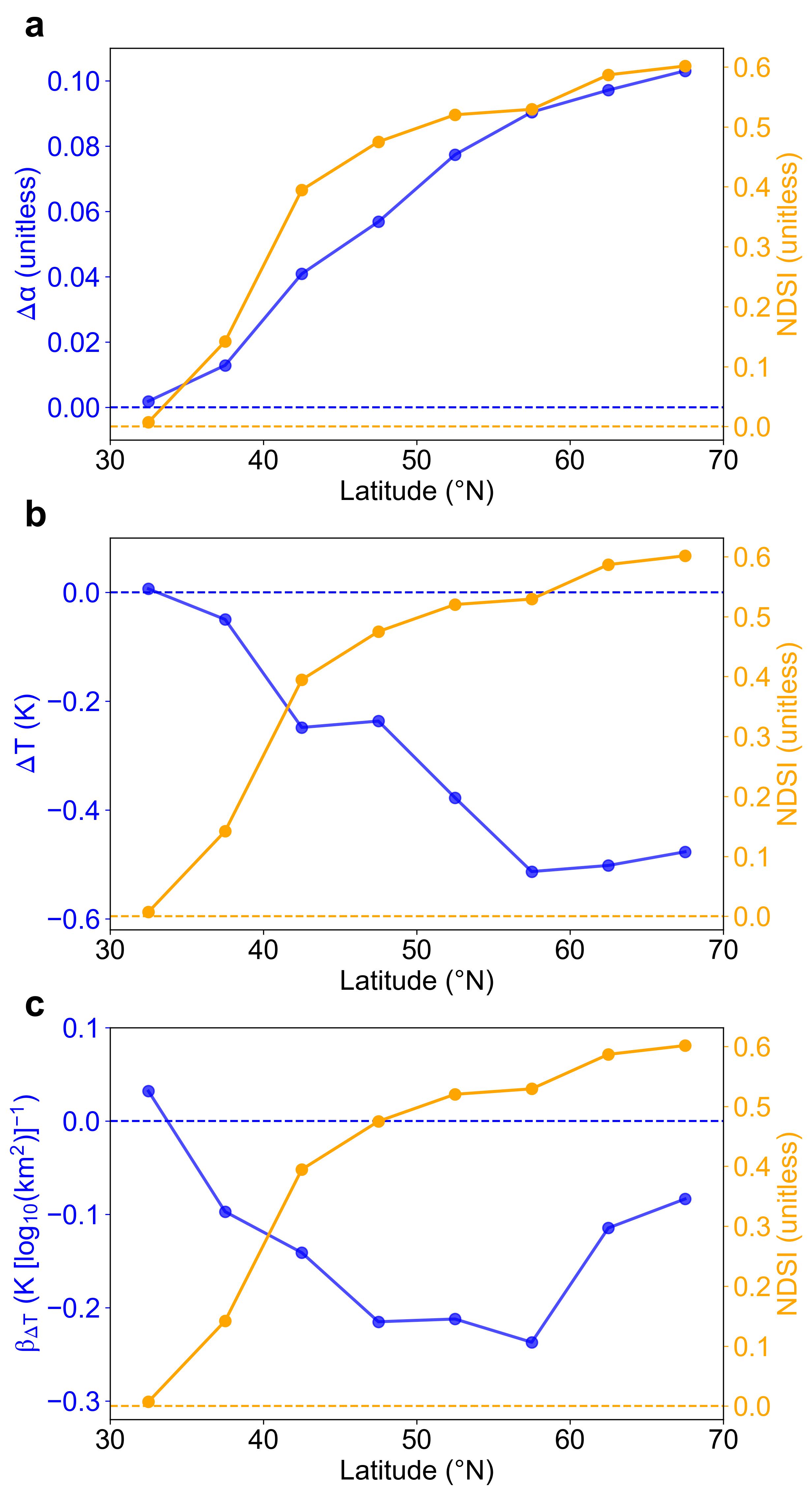


**Supplementary Figure 14 | Latitudinal changes in snow cover and winter (December–February) biogeophysical changes one year after fire.** The study domain is North America from 30ºN to 70ºN. The Global Fire Atlas dataset, covering the years 2003 to 2016, was used. The snow cover extent measured by MODIS Normalized Difference Snow Index (NDSI) is shown in yellow in all three panels using the right-hand vertical axis. Postfire changes in winter surface albedo (Δα) (**a**), surface temperature change (ΔΤ) (**b**) and fire-size sensitivity (β_ΔΤ_) (**c**) are shown. Linear regressions models (ΔΤ = α + β_ΔΤ_ × log_10_(fire size)) were fitted using original fire patches across different latitudinal bands with 5° intervals, with significant regressions being shown in filled circles (*α*=0.05, the student’s t-test). Figure developed using the Python open-source tools.


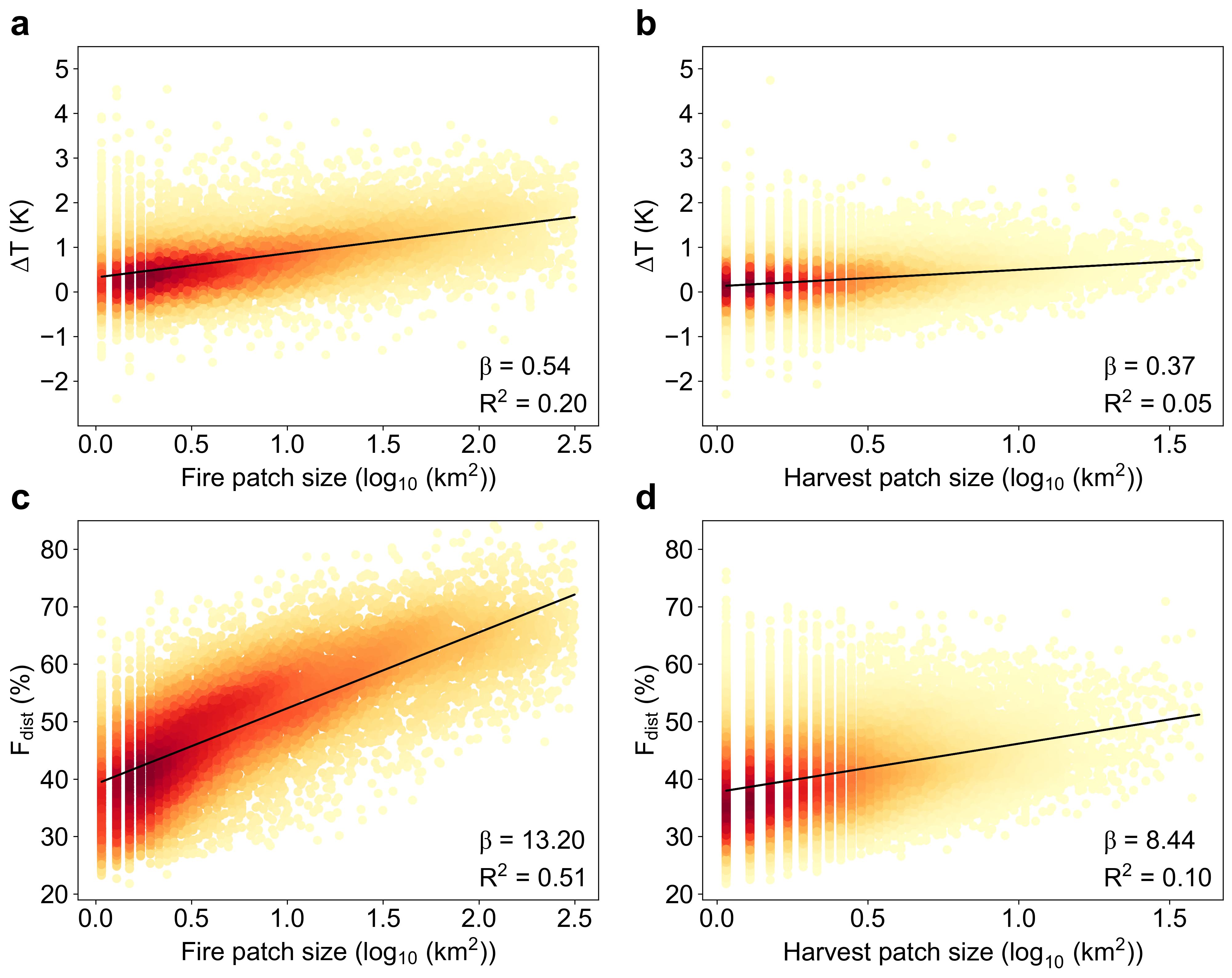


**Supplementary Figure 15 | Relationships between summer (June–August) land surface temperature change one year after disturbance (ΔΤ), (a, b), disturbance fraction (F_dist_, percent) (c, d) and patch size following fire (a, c) or forest harvest (b, d) for Canadian forests**. F_dist_ is calculated as the ratio between the number of 30m pixels subject to stand-replacing fire or clearcut harvest and the total number of 30m pixels in a given patch of fire or harvest. The regression slope (β) and the coefficient of determination (R^2^) were derived by fitting a linear regression model (y = α + β× log_10_(patch size)), where y stands for ΔΤ and F_dist_. All regressions were significant (*p*<0.05, the student’s t-test). Figure developed using the Python open-source tools.


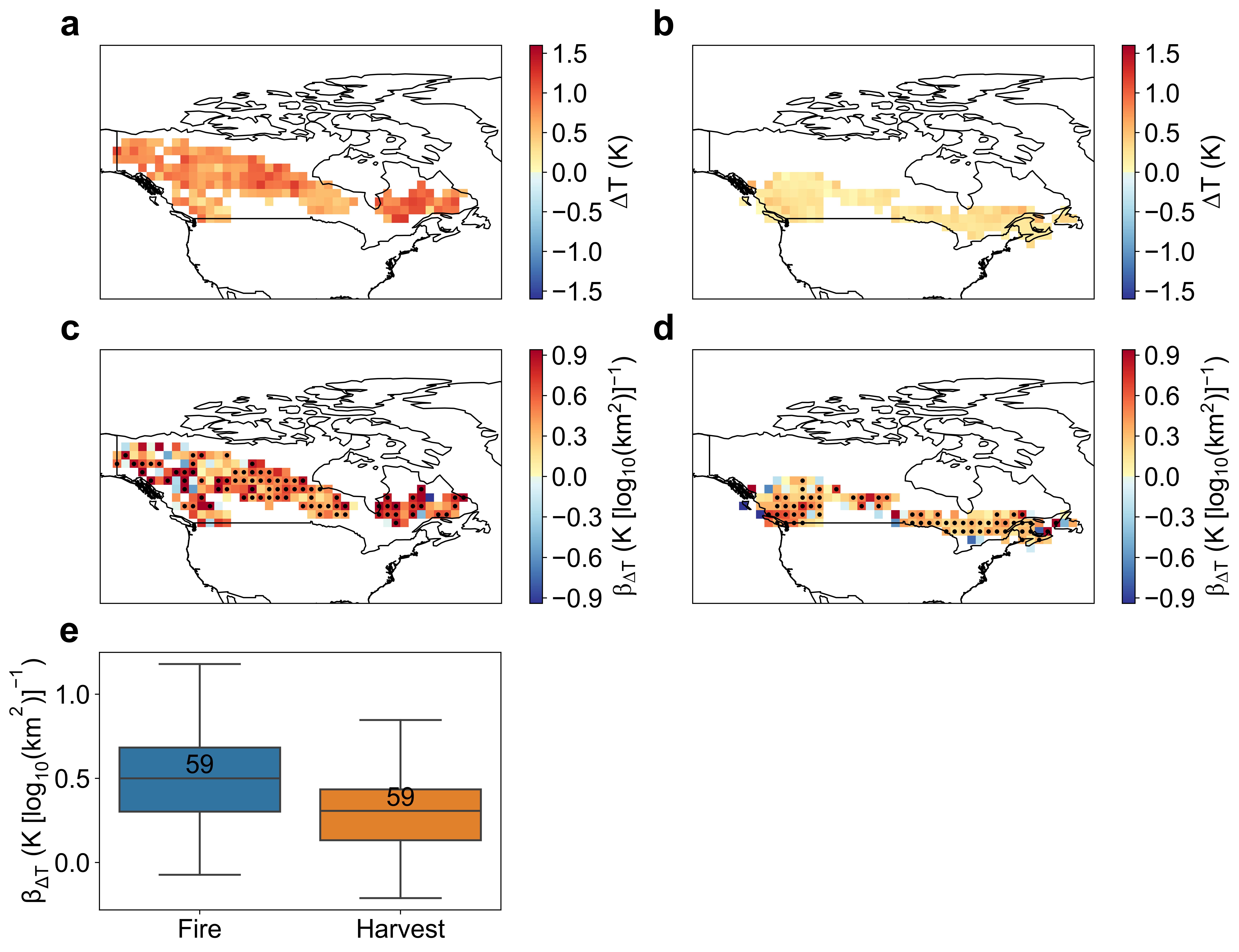


**Supplementary Figure 16 | Changes in summer (June–August) land surface temperature one year after disturbance (ΔΤ) and their amplification by patch size following fire or forest harvest across Canadian forests**. Panels (**a**) and (**b**) show the mean ΔΤ following fire disturbance (**a**) or forest harvest (**b**) for each 2º grid cell. Multiple linear regression models (y = α + β_ΔΤ_ × log_10_(patch size) + β_F_ × F_dist_) were fitted to derive the partial effect of patch size on ΔT (β_ΔT_), after accounting for F_dist_, for fire disturbance (**c**) and forest harvest (**d**) for each 2º grid cell with more than 10 disturbance patches bigger than 1 km^2^. Solid dots indicate pixels with locally significant regressions (*p*<0.05, the two-tailed t-test). All significant regressions have passed a more rigorous field significance test corrected for the false discovery rate (α_FDR_ = 0.10, see Methods). (**e**) Comparison of β_ΔT_ between fire and harvest. The center line of the boxplots represents the median value, with box limits indicating upper and lower quartiles and whiskers showing 1.5 × interquartile range. Figure developed using the Python open-source tools.


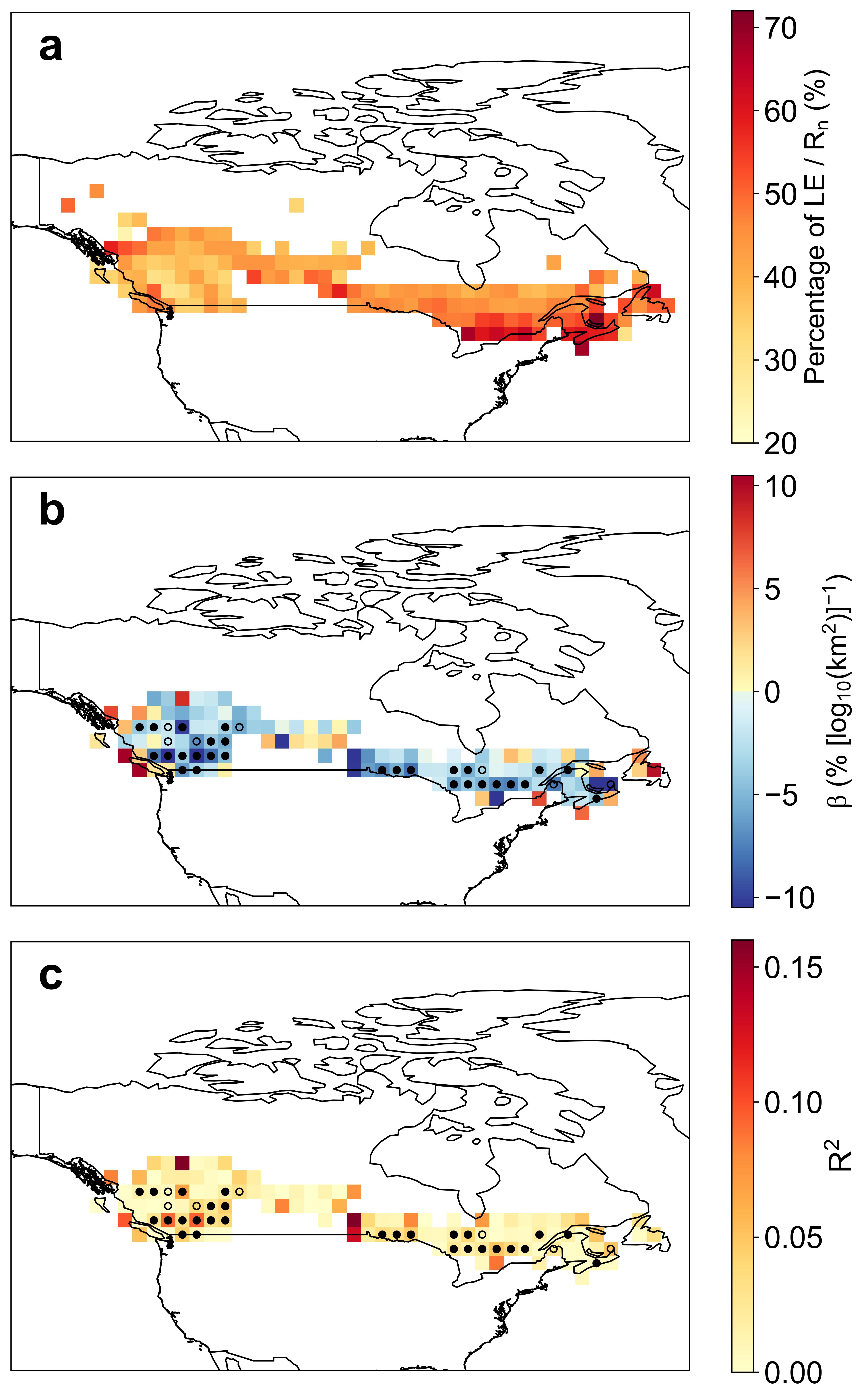


**Supplementary Figure 17 | The percentage (%) of latent heat flux to net radiation (LE/R_n_) for summer (June–August) one year after harvest and its change with harvest patch size across Canadian forests.** Panel (**a**) displays the mean values of percentage of LE/R_n_ for 2º grid cells. Panels (**b**) and (**c**) show the regression slope (β) and the coefficient of determination (R^2^) derived by fitting a linear regression model (y = α + β × log_10_(patch size)) within the 2º grid cells with more than 10 harvest events, where y represents the percentage of LE/R_n_. Both solid and empty dots indicate pixels with locally significant regressions (*p*<0.05, the two-tailed t-test), but solid dots indicate those having passed a more rigorous field significance test corrected for the false discovery rate (α_FDR_ = 0.10, see Methods). Figure developed using the Python open-source tools.


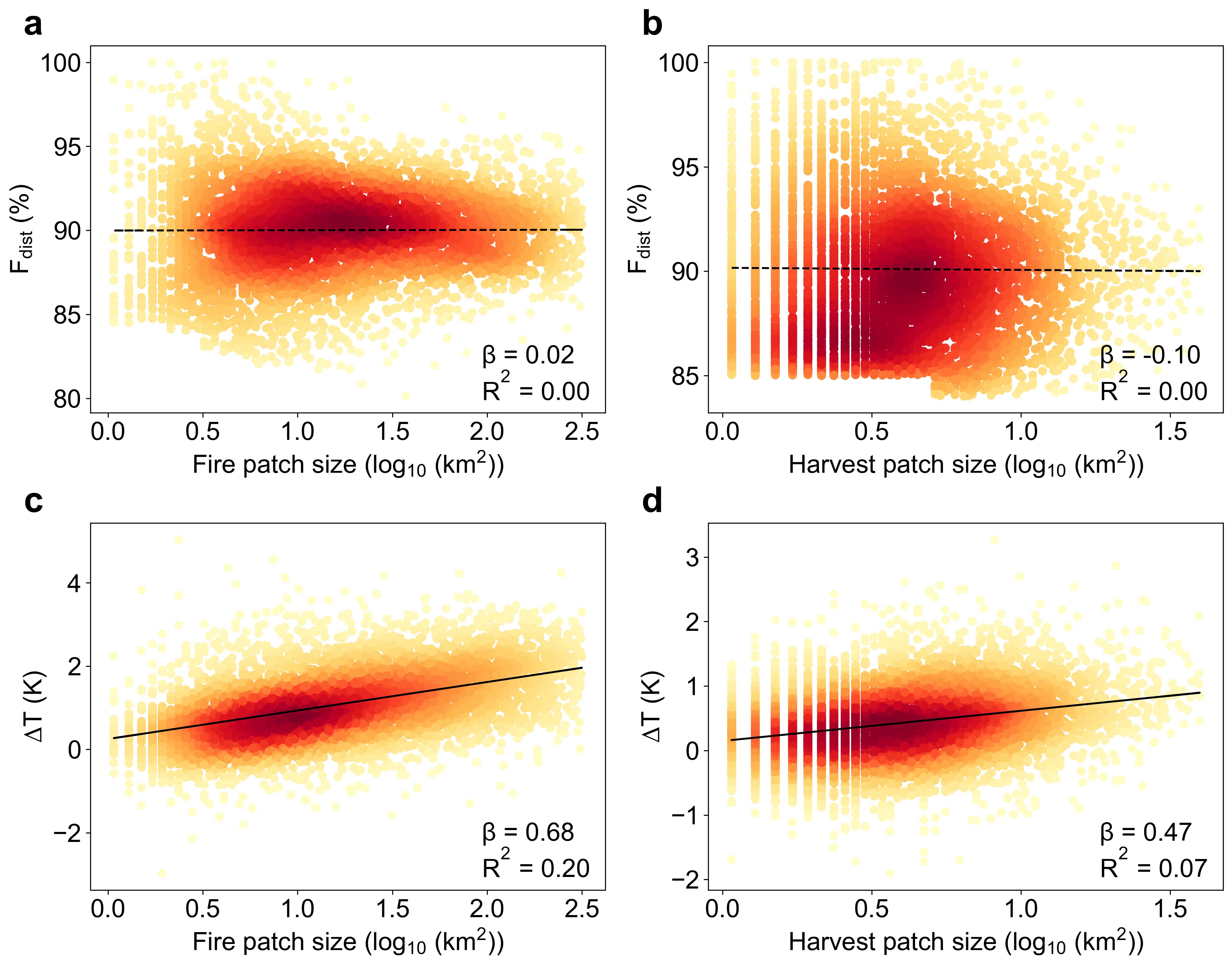


**Supplementary Figure 18 | Relationships between summer (June–August) land surface temperature change one year after disturbance (ΔΤ) and patch size for fire and forest harvest across Canadian forests, after manually maintaining a relatively unchanged disturbance fraction (F_dist_) when disturbance patch size increases.** F_dist_ is calculated as the ratio between the number of 30m pixels subject to stand-replacing fire or clearcut harvest and the total number of 30m pixels in a given patch of fire or harvest. By selecting appropriate F_dist_ threshold values (Supplementary Table 4) for patches of different size bins (see Supplementary Table 4 for details), a constant F_dist_ of approximately 90% was maintained across different patch sizes for both fire (**a**) and harvest (**b**). The regression slope (β) and the coefficient of determination (R^2^) were derived by fitting a linear regression model (y = α + β× log_10_(patch size)), where y stands for ΔΤ (**c** for fire, **d** for harvest) and F_dist_ (**a** for fire, **b** for harvest). Solid lines indicate significant regressions (*p*<0.05); dashed lines indicate insignificant regressions (*p*>0.05). Figure developed using the Python open-source tools.


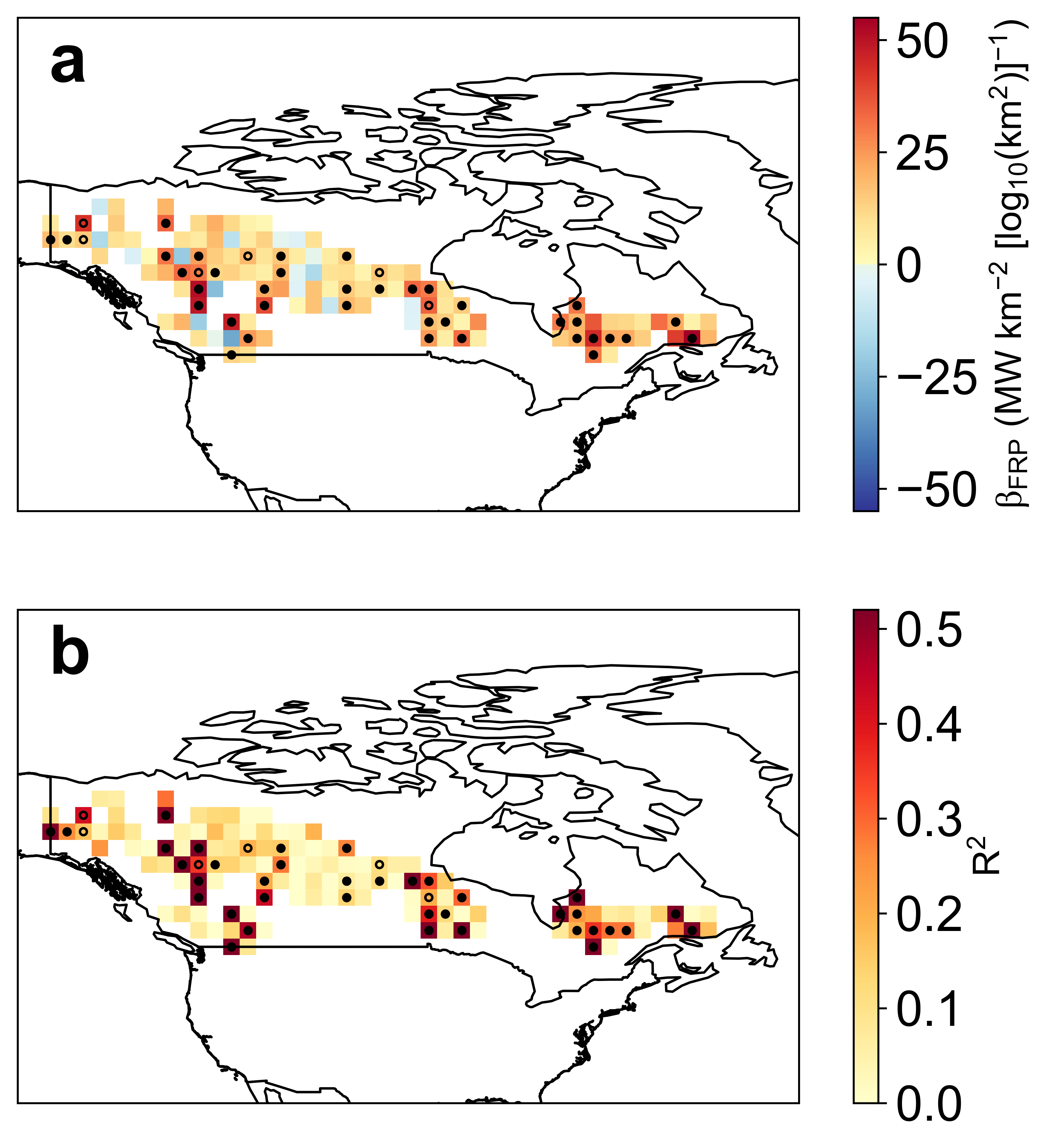


**Supplementary Figure 19 | Relationships between fire radiative power and fire size across Canadian forests at a 2º resolution.** Panels (**a**) and (**b**) show the linear regression and the coefficient of determination (R^2^) derived by fitting a linear regression model (FRP = α + β × log_10_(fire size)). Only grid cells with >10 fire events larger than 1 km^2^ were included in the regression analysis. Both solid and empty dots indicate pixels with locally significant regressions (*p*<0.05, the two-tailed t-test), but solid dots indicate those having passed a more rigorous field significance test corrected for the false discovery rate (α_FDR_ = 0.10, see Methods). Figure developed using the Python open-source tools.


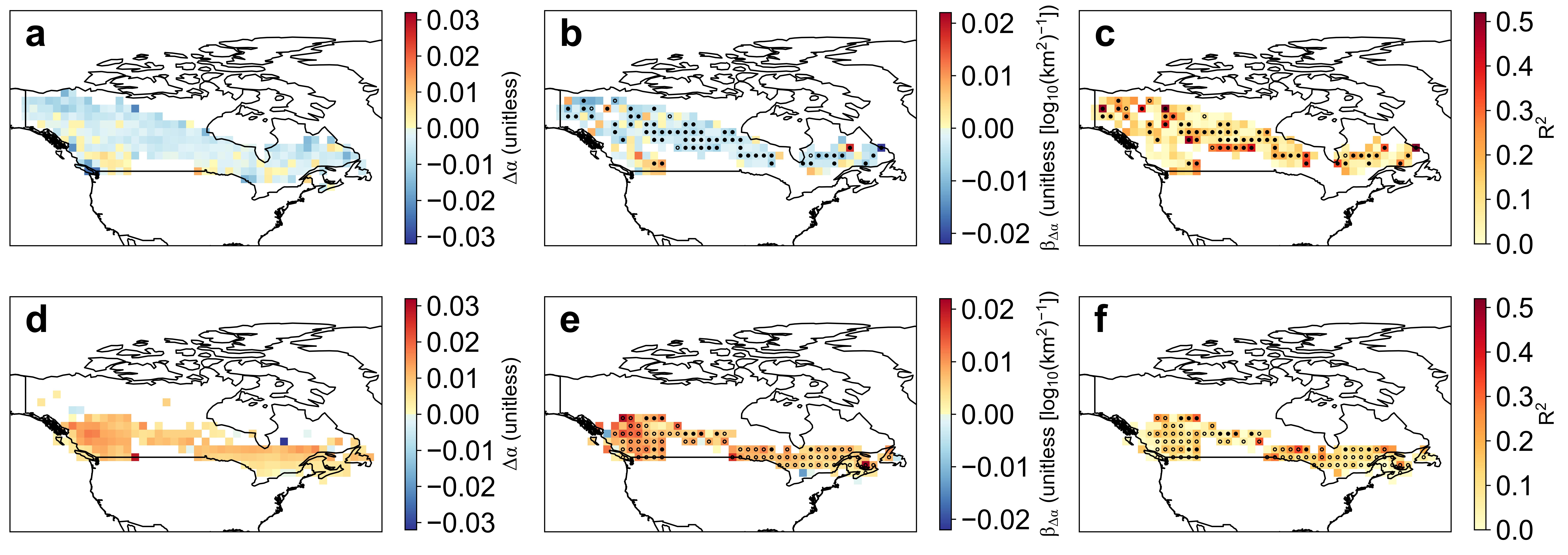


**Supplementary Figure 20 | Disturbance patch size impact on summer (June–August) albedo change (Δα) one year after disturbance in Canada.** The first (**a**–**c**) and second (**d**–**f**) rows of panels relate to fire and harvest, respectively. The first column displays the mean Δα for each 2º grid cell. The second and third columns show the linear regression (β_Δα_) and the coefficient of determination (R^2^) derived by fitting a linear regression model (Δα = intercept + β_Δα_ × log_10_(patch size)). Only grid cells with >10 disturbance events larger than 1 km^2^ were included in the regression analysis. Both solid and empty dots indicate pixels with locally significant regressions (*p*<0.05, the two-tailed t-test), but solid dots indicate those having passed a more rigorous field significance test corrected for the false discovery rate (α_FDR_ = 0.10, see Methods). Figure developed using the Python open-source tools.


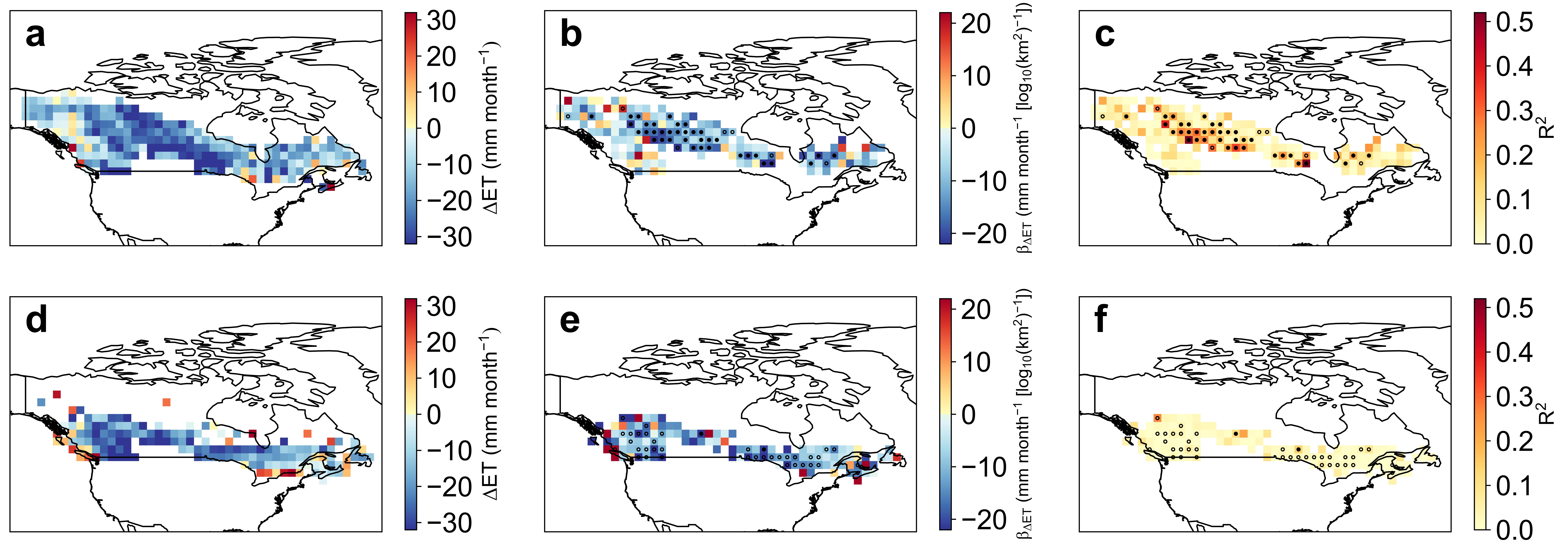


**Supplementary Figure 21 | Disturbance patch size impact on summer (June–August) evapotranspiration change (ΔET) one year after disturbance in Canada.** The first (**a**–**c**) and second (**d**–**f**) rows of panels relate to fire and harvest, respectively. The first column displays the mean ΔET for each 2º grid cell. The second and third columns show the slope (β_ΔET_) and the coefficient of determination (R^2^) derived by fitting a linear regression model (ΔET = α + β_ΔET_ × log_10_(patch size)). Only grid cells with >10 disturbance events larger than 1 km^2^ were included in the regression analysis. Both solid and empty dots indicate pixels with locally significant regressions (*p*<0.05, the two-tailed t-test), but solid dots indicate those having passed a more rigorous field significance test corrected for the false discovery rate (α_FDR_ = 0.10, see Methods). Figure developed using the Python open-source tools.


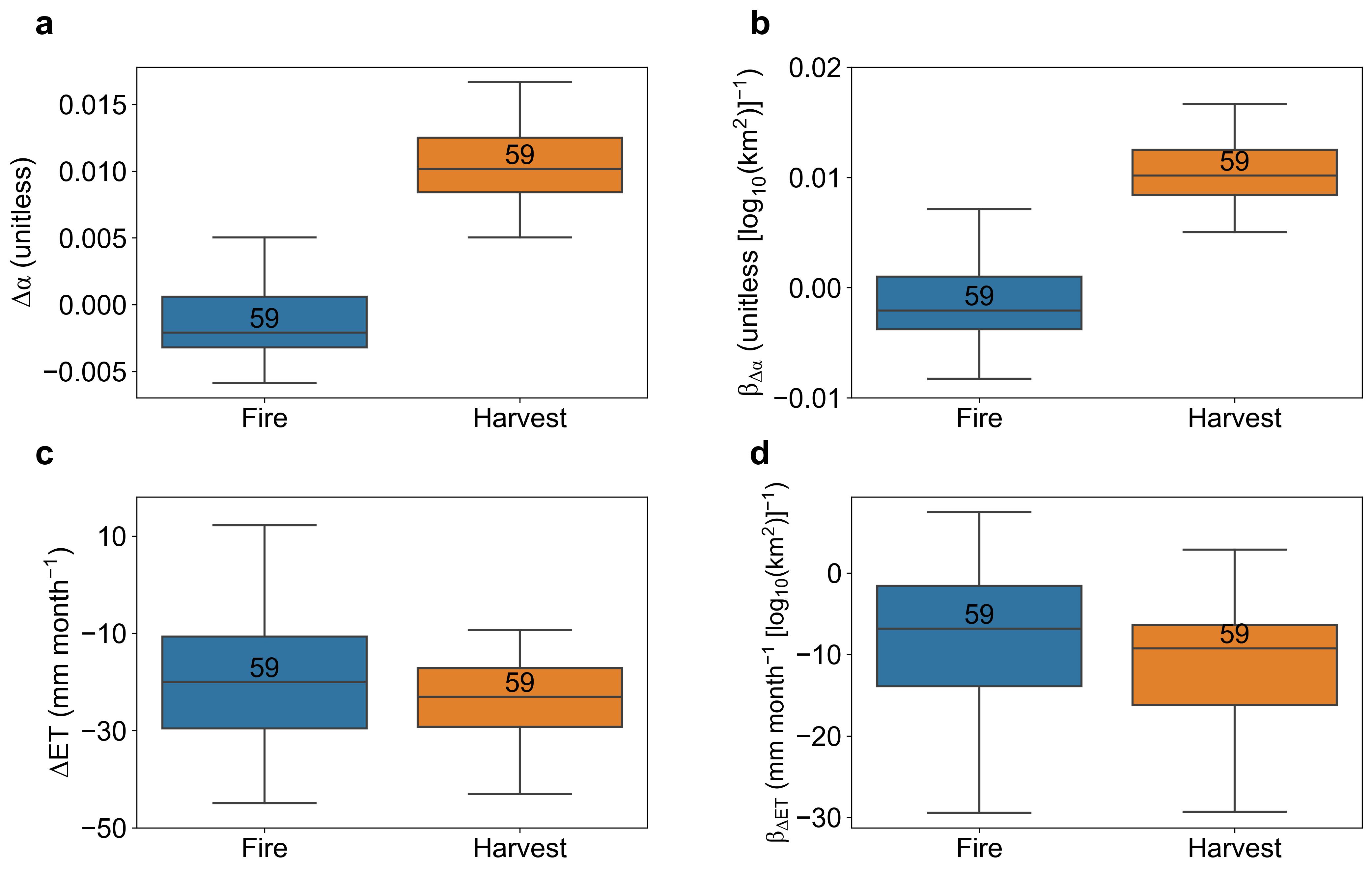


**Supplementary Figure 22 | Comparison of post-disturbance changes in surface albedo and evapotranspiration and their amplification with patch size for fire and harvest.** Boxplots are shown for changes in surface albedo (Δα, **a**) and evapotranspiration (ΔEΤ, **c**) in summer (June–August) one year after disturbance. Simple linear regression models (y = α + β × log_10_(patch size)) were fitted to derive the amplification effect (β) of fire size on Δα (β_Δα_, **b**) and ΔET (β_ΔET_, **d**) for each 2º grid cell with more than 10 fires bigger than 1 km^2^ (Supplementary Fig. 20 and 21). The spatial domain of the 2º grid used to derive the boxplots covers the overlapping areas (n=59) of both forest fire and harvest. The center line of the boxplots represents the median value, with box limits indicating upper and lower quartiles and whiskers showing 1.5 × interquartile range. Figure developed using the Python open-source tools.


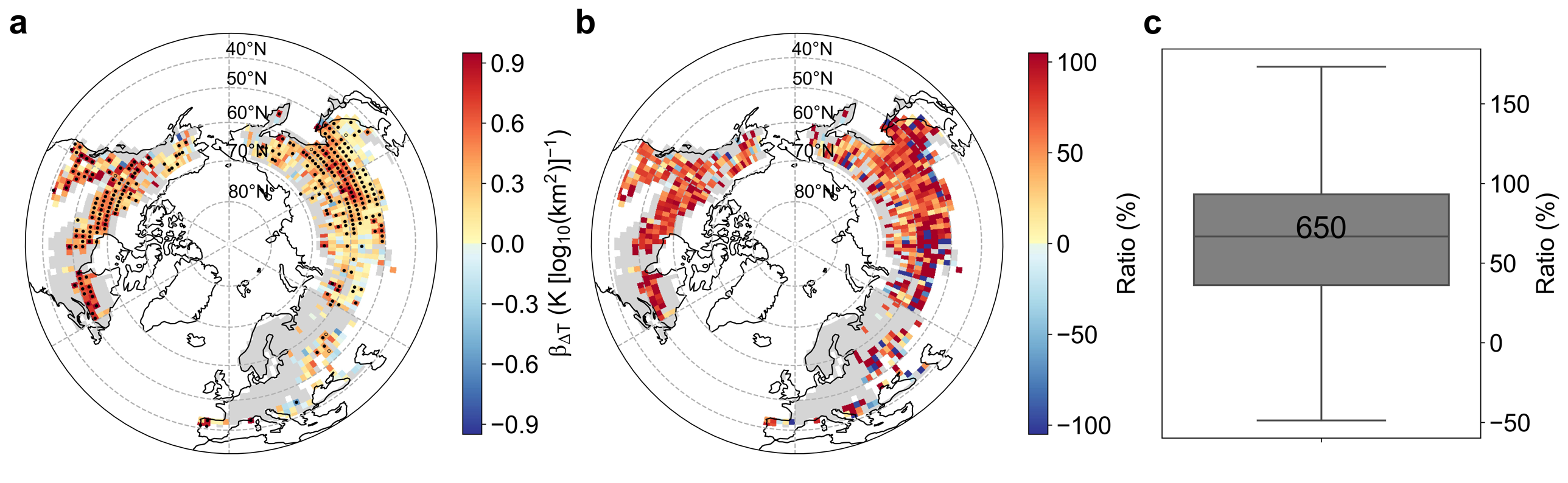


**Supplementary Figure 23 | The spatial distribution of the partial coefficient of fire size to postfire change in surface radiometric temperature change in summer (June–August) one year after fire.** (**a**) The partial β_ΔΤ_ was derived from multiple linear regressions using summer ΔT as the dependent variable, and log_10_(fire size), ΔLAI, FRP and forest mortality as independent variables, which were performed for each 2º grid cell. Both solid and empty dots indicate pixels with locally significant regressions (*p*<0.05, the two-tailed t-test), but solid dots indicate those having passed a more rigorous field significance test corrected for the false discovery rate (α_FDR_ = 0.10, see Methods). (**b**) Ratio of partial β_ΔΤ_ to that derived by a simple linear regression (ΔΤ = α + β_ΔΤ_ × log_10_(fire size)) and its boxplot (**c**). The center line of the boxplot (n=650) represents the median value, with box limits indicating upper and lower quartiles and whiskers showing 1.5 × interquartile range. Figure developed using the Python open-source tools.


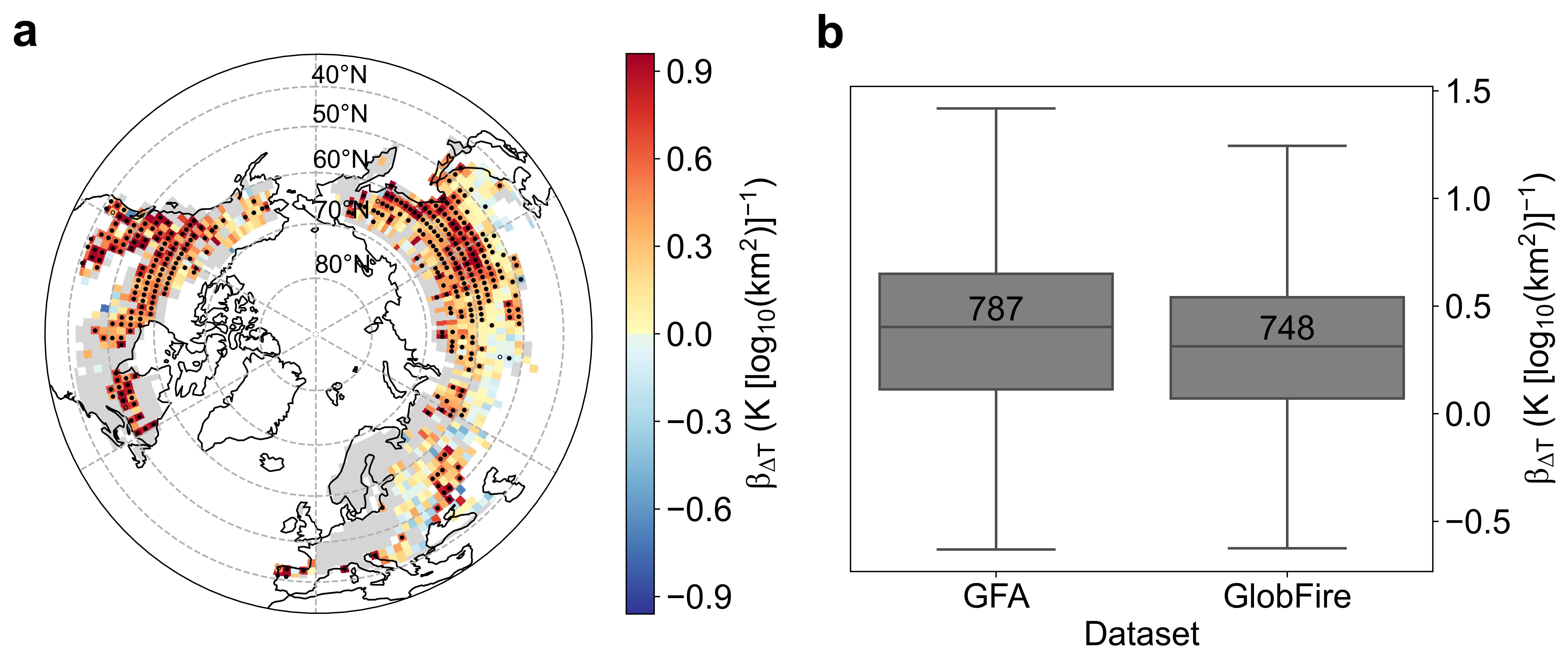
**Supplementary Figure 24 |** **Spatial pattern of the regression slope (β_ΔT_) between summer (June–August) surface warming one year after fire and fire size based on the GlobFire dataset.** Panel (**a**) displays the β_ΔΤ_ derived by fitting a linear regression model (ΔΤ = α + β_ΔΤ_ × log_10_(fire size)) within the 2º grid cells containing more than 10 fires. Both solid and empty dots indicate pixels with locally significant regressions (*p*<0.05, the two-tailed t-test), but solid dots indicate those having passed a more rigorous field significance test corrected for the false discovery rate (α_FDR_ = 0.10, see Methods). The light grey background indicates northern forests with a >10% ground coverage. For comparison, panel (**b**) also show the β_ΔT_ derived from the Global Fire Atlas (GFA) dataset. The center line of the boxplot represents the median value, with box limits indicating upper and lower quartiles and whiskers showing 1.5 × interquartile range. The number above each box indicates the count of grid cells. Figure developed using the Python open-source tools.


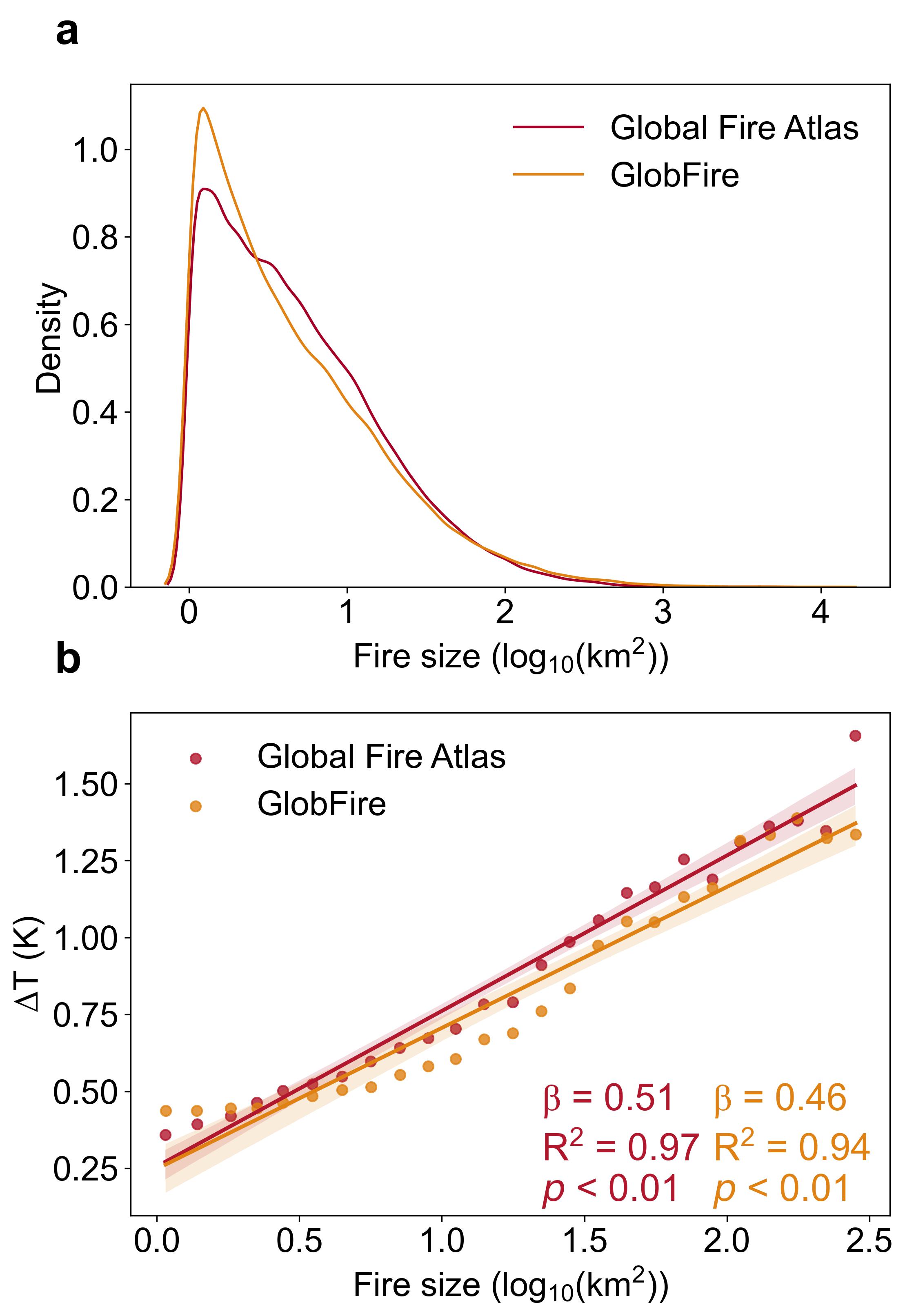


**Supplementary Figure 25 |** **Structure of forest fire size according to the Global Fire Atlas (GFA, red) and the GlobFire (orange) datasets during 2003–2016.** (**a**) Probability density distribution of fire size. (**b**) The linear relationship between postfire summer (June–August) ΔT and fire size (β_ΔT_) derived from the GFA and GlobFire dataset through grouping fire patches into bins of different sizes. Fire events were grouped into different bins with intervals of 0.1 in the logarithmic scale, with the mean summer ΔT being derived for each bin. β and R^2^ represent the linear regression slope and the coefficient of determination, respectively (n=25, with the significance test being made using the student’s t-test). Shading denotes 95% prediction intervals. A linear regression model (ΔT = α + β × log_10_(fire size)) was also performed using original fire patches without binning, with β values for Global Fire Atlas and GlobFire being 0.44 (*p*<0.01, R²=0.08) and 0.34 (*p*<0.01, R²=0.05), respectively. Figure developed using the Python open-source tools.


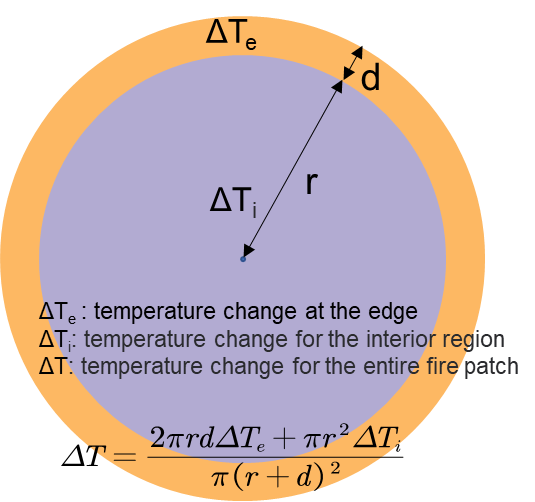


**Supplementary Figure 26 | Derivation of the postfire land surface temperature change for the entire fire patch (ΔT) from those in the interior (ΔT_i_) and at the edge (ΔT_e_) using a simple theoretical model**. Here, *d* is chosen as 500m (i.e., a single MODIS pixel).


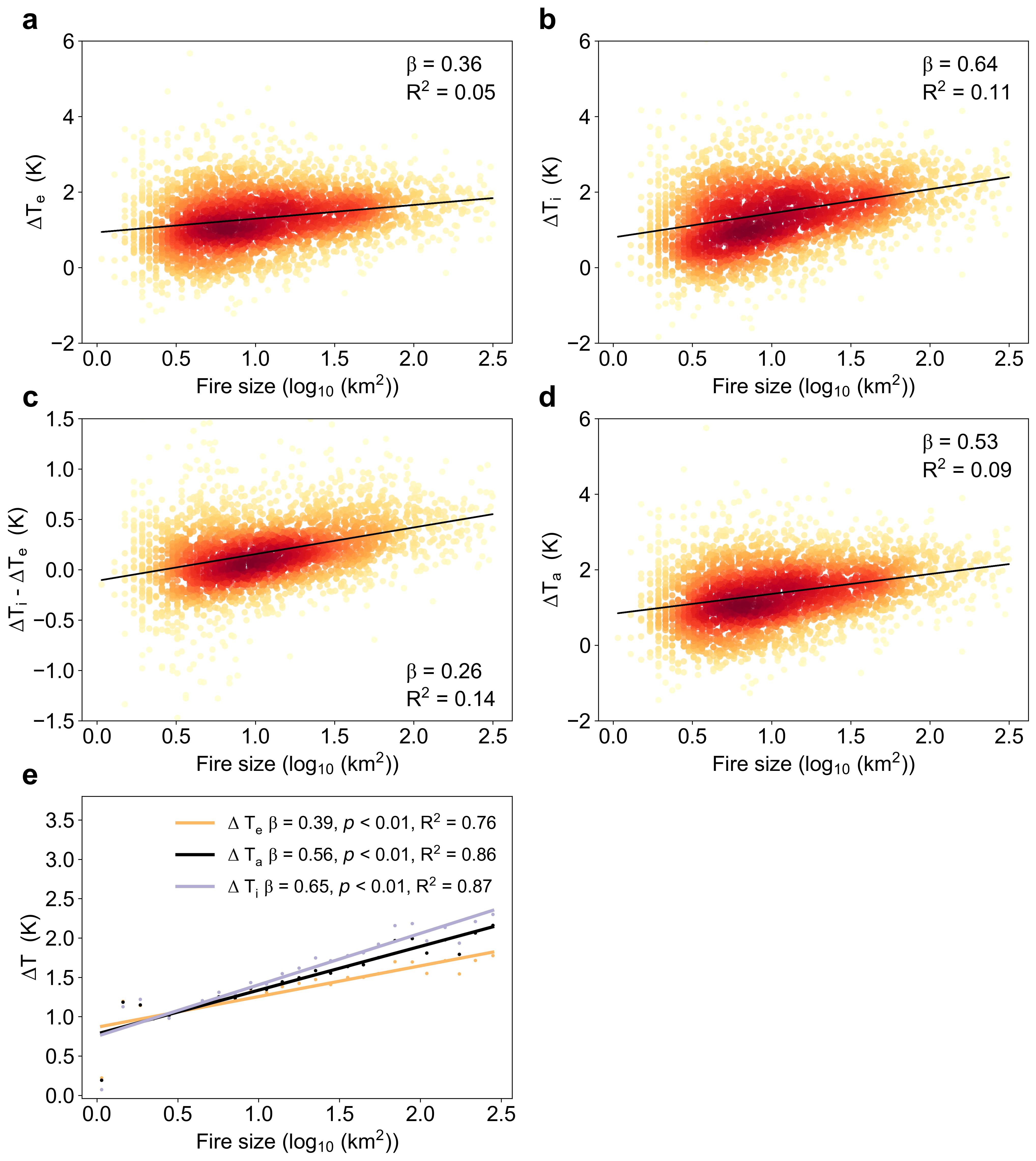


**Supplementary Figure 27 | The relationships between postfire land surface temperature change (ΔT) for the entire fire patch, for the interior and for the edge and fire size in US Alaska and Canada** **for 2003–2006 using the Global Fire Atlas dataset.** (**a**) ΔT for the edge (ΔT_e_); (**b**) ΔT for the interior within patches (ΔT_i_); (**c**) the difference between ΔT_i_ and ΔT_e_. (**d**) ΔT for the whole patch by averaging ΔT_i_ and ΔT_e_. (**e**) Changes in ΔT_i_, ΔT_e_ and ΔT_a_ in summer (June–August) with increasing fire size based on regressions with binning. Fire events were grouped into different bins with intervals of 0.1 in the logarithmic scale, with the mean ΔT being derived for each bin. β and R^2^ represent the linear regression slope and the coefficient of determination, respectively (n=25, with the significance test being made using the student’s t-test). Figure developed using the Python open-source tools.





**Supplementary Figure 28 | The relationships between changes in daytime (a**–**c), daily (d**–**f) and nighttime (g**–**i) surface radiometric temperature** **(ΔΤ) and fire size in summer (****June–August) one year after fire across northern forests (40ºN–70ºN).** The first column of panels displays the mean ΔΤ for each 2º grid cell. The second and third columns show the regression slope (β_ΔΤ_) and the coefficient of determination (R^2^), respectively, derived by fitting a linear regression model (ΔΤ = α + β_ΔΤ_ × log_10_(fire size)) within the 2º grid cells containing more than 10 fires. Both solid and empty dots indicate pixels with locally significant regressions (*p*<0.05, the two-tailed t-test), but solid dots indicate those having passed a more rigorous field significance test corrected for the false discovery rate (α_FDR_ = 0.10, see Methods). The light grey background in all maps indicates northern forests with a >10% ground coverage. Figure developed using the Python open-source tools.

**Supplementary tables**

Supplementary Table 1 | Data sources for historical burned area and the number of fires.

| Country | State | Dataset | Agency | Period | Sources |
| --- | --- | --- | --- | --- | --- |
| Canada | - | National Fire Database | Natural Resources Canada’s Canadian Wildland Fire Information System | 1960-2020 | https://cwfis.cfs.nrcan.gc.ca/datamart |
| USA | - | Monitoring Trends in Burn Severity (MTBS) Burned Areas Boundaries Dataset | U.S. Forest Service, Remote Sensing Applications Center and the U.S. Geological Survey, Earth Resources Observation and Science Center | 1984-2020 | https://www.mtbs.gov/ |
| Australia | New South Wales | National Parks & Wildlife Service (NPWS) Fire History - Wildfires and Prescribed Burns | Department of Planning, Industry and Environment | 1981-2020 | https://datasets.seed.nsw.gov.au/dataset/fire-history-wildfires-and-prescribed-burns-1e8b6 |
|  | Western Australia | Department of Biodiversity, Conservation and Attractions (DBCA) Fire History (DBCA-060) | Department of Biodiversity, Conservation and Attractions | 1981-2020 | https://catalogue.data.wa.gov.au/dataset/dbca-fire-history |
|  | South Australia | Bushfires and Prescribed Burns History | Department for Environment and Water | 1981-2020 | https://data.sa.gov.au/data/dataset/fire-history |
|  | Victoria | Fire History Records of Fires primarily on Public Land | Department of Environment, Land, Water & Planning | 1981-2020 | https://discover.data.vic.gov.au/dataset/fire-history-records-of-fires-primarily-on-public-land |
|  | Queensland | Wildfire History — SHP | Brisbane City Council | 1981-2020 | https://www.data.brisbane.qld.gov.au/data/dataset/wild-fire-history/resource/2e8c7996-b864-4166-a1a0-953370ab63e5?inner_span=True |
|  | Tasmania | List Fire History – Statewide Coverage | Department of Primary Industries, Parks, Water and Environment | 1981-2020 | http://listdata.thelist.tas.gov.au/opendata/ |

**Supplementary Table 2 |** **Comparison of winter biogeophysical changes one year after fire and snow cover between northern temperate and boreal forests and Australian forests for 2003–2016.** For the northern temperate and boreal regions, summer is defined as June to August, and winter is defined as December to February of the following year. For Australia, summer is defined as December to February of the following year, and winter is defined as June to August. Snow cover is measured by MODIS Normalized Difference Snow Index (NDSI) which ranges between 0 (almost no snow) to 1 (almost full snow cover). The number before “±” represents the mean value, and the number after “±” represents the standard deviation.

|  | Northern temperate and boreal forests (40ºN–70ºN) | Australia |
| --- | --- | --- |
| NDSI (unitless) | 0.48 ± 0.13 | 0.00009 ± 0.001 |
| Δα (unitless) | 0.03 ± 0.06 | 0.0009 ± 0.009 |
| ΔET (mm month^-1^) | -0.20 ± 1.18 | -0.69 ± 6.45 |
| ΔT (K) | -0.20 ± 0.83 | 0.10 ± 0.41 |

**Supplementary Table 3 |** **Statistical analysis** **of the relationship between summer (June–August) land surface temperature change one year after forest fire or harvest in Canadian forests.** The regression model takes the form ‘ΔΤ ~ log_10_(size)*Type+F_dist_*Type’, where ΔΤ is summer land surface temperature change following forest disturbance, log_10_(size) is the logarithm of disturbance patch size, ‘Type’ is a categorical variable indicating the disturbance type (i.e., fire or harvest), F_dist_ is the disturbance fraction for a given patch, and the ‘*’ sign represents the interactive effect. The model has a sample size of 39640, with an overall p-value<0.001 and an R^2^ of 0.23.

|  | Disturbance type | Estimated coefficient | Standard error | p-value | p-value for the difference between harvest and fire |
| --- | --- | --- | --- | --- | --- |
| Log_10_(size) | fire | 0.494 | 0.017 | <0.01 | <0.01 |
|  | harvest | 0.327 | 0.011 | <0.01 |  |
| F_dist_ | fire | 0.362 | 0.082 | <0.01 | 0.14 |
|  | harvest | 0.483 | 0.041 | <0.01 |  |

**Supplementary Table 4 | F_dist_ threshold values used for inclusion of the 500m pixels in order to maintain a constant F_dist_ (around 90%) across different patch sizes.** To maintain a consistent F_dist_ across different sizes of disturbance patches, disturbance events were grouped into bins with intervals of 0.1 in terms of log_10_(size) to search for the appropriate F_dist_ threshold values for each size bin. For this purpose, the average F_dist_ value for all patches within each size bin was calculated using all possible F_dist_ threshold values, at 0.5% intervals, between 70% and 99.5%, and the threshold value giving an average F_dist_ for a given size bin closest to 90% was selected and shown.

| Log_10_(size) | F_dist_ threshold value (%) | |
| --- | --- | --- |
|  | Fire | Harvest |
| (0.0,0.1) | 84.5 | 85.5 |
| (0.1,0.2) | 85.5 | 85.0 |
| (0.2,0.3) | 84.5 | 84.5 |
| (0.3,0.4) | 84.0 | 84.5 |
| (0.4,0.5) | 84.0 | 84.5 |
| (0.5,0.6) | 83.0 | 85.0 |
| (0.6,0.7) | 82.5 | 84.5 |
| (0.7,0.8) | 82.0 | 84.0 |
| (0.8,0.9) | 82.0 | 84.0 |
| (0.9,1.0) | 82.0 | 84.0 |
| (1.0,1.1) | 81.5 | 84.0 |
| (1.1,1.2) | 81.5 | 84.5 |
| (1.2,1.3) | 80.5 | 84.0 |
| (1.3,1.4) | 80.0 | 84.5 |
| (1.4,1.5) | 80.0 | 84.0 |
| (1.5,1.6) | 79.5 | 82.0 |
| (1.6,1.7) | 79.5 |  |
| (1.7,1.8) | 78.5 |  |
| (1.8,1.9) | 78.5 |  |
| (1.9,2.0) | 78.0 |  |
| (2.0,2.1) | 77.0 |  |
| (2.1,2.2) | 77.0 |  |
| (2.2,2.3) | 77.0 |  |
| (2.3,2.4) | 77.0 |  |
| (2.4,2.5) | 75.5 |  |

**Supplementary Table 5 |** **Statistical analysis** **of the relationship between summer (June–August) land surface temperature change one year after fire and fire size while accounting for co-varying FRP and disturbance fraction in Canadian forests.** The regression models take sequential forms: ΔΤ ~ log_10_(size); ΔΤ ~ log_10_(size)+ F_dist_; and ΔΤ ~ log_10_(size) +F_dist_+FRP; where ΔΤ is summer land surface temperature change one year after fire, log_10_(size) is the logarithm of fire size, FRP is fire radiative power indicating fire intensity, and F_dist_ is the disturbance fraction for a given fire patch. The numbers within the brackets indicate the standard error.

| Regression Equation | Log_10_(size) | | F_dist_ | | FRP | | Intercept | | Overall | R^2^ | Sample |
| --- | --- | --- | --- | --- | --- | --- | --- | --- | --- | --- | --- |
|  | Estimate | p-value | Estimate | p-value | Estimate | p-value | Estimate | p-value | p-value |  | size |
| ΔT~log_10_(size) | 0.588(0.017) | <0.01 |  |  |  |  | 0.493(0.021) | <0.01 | <0.01 | 0.23 | 3728 |
| ΔT~log_10_(size)+F_dist_ | 0.553(0.025) | <0.01 | 0.287(0.144) | <0.05 |  |  | 0.375(0.063) | <0.01 | <0.01 | 0.23 | 3728 |
| ΔT~log_10_(size)+F_dist_+FRP | 0.518(0.024) | <0.01 | 0.212(0.141) | 0.13 | 0.004(0.000) | <0.01 | 0.314(0.062) | <0.01 | <0.01 | 0.26 | 3728 |

**Supplementary Table 6 | Linear regressions of summer (June–August) land use temperature change one year after fire, by progressively incorporating different explanatory variables over northern forests of 40ºN–70ºN in a linear regression framework.** The numbers within the brackets indicate standard errors.

| Regression form | Log_10_(size) | | Forest mortality (0-1) | | FRP | | ΔLAI | | Intercept | | Overall | R^²^ | Sample |
| --- | --- | --- | --- | --- | --- | --- | --- | --- | --- | --- | --- | --- | --- |
|  | Estimate | p-value | Estimate | p-value | Estimate | p-value | Estimate | p-value | Estimate | p-value | p-value |  | size |
| ΔT~Log_10_(size) | 0.383(0.007) | <0.01 |  |  |  |  |  |  | 0.562(0.007) | <0.01 | <0.01 | 0.06 | 49590 |
| ΔT~Log_10_(size)+mortality | 0.289(0.005) | <0.01 | 1.652(0.009) | <0.01 |  |  |  |  | 0.002(0.006) | 0.78 | <0.01 | 0.46 | 49590 |
| ΔT~Log_10_(size)+mortality+FRP | 0.272(0.005) | <0.01 | 1.621(0.009) | <0.01 | 0.002(0.000) | <0.01 |  |  | -0.035(0.006) | <0.01 | <0.01 | 0.46 | 49590 |
| ΔT~Log_10_(size)+ΔLAI+FRP | 0.292(0.006) | <0.01 |  |  | 0.004(0.000) | <0.01 | -0.657(0.005) | <0.01 | 0.084(0.007) | <0.01 | <0.01 | 0.32 | 49590 |
| ΔT~Log_10_(size)+mortality+FRP+ΔLAI | 0.269(0.005) | <0.01 | 1.392(0.012) | <0.01 | 0.002(0.000) | <0.01 | -0.179(0.006) | <0.01 | -0.067(0.006) | <0.01 | <0.01 | 0.47 | 49590 |

Supplementary Table 7 | Data sources for expenditure on forest fire control in Canada and the USA.

| **Country** | **Agency** | **Period** | **Data sources** |
| --- | --- | --- | --- |
| Canada | The official website of the Government of Canada | 1970–2017 | https://www.nrcan.gc.ca/climate-change/impacts-adaptations/climate-change-impacts-forests/forest-change-indicators/cost-fire-protection/17783#how |
| USA | National Interagency Fire Center | 1985–2021 | https://www.nifc.gov/fire-information/statistics/suppression-costs |
